# *C9orf72* FTLD/ALS-associated Gly-Ala dipeptide repeat proteins cause neuronal toxicity and Unc119 sequestration

**DOI:** 10.1007/s00401-014-1329-4

**Published:** 2014-08-14

**Authors:** Stephanie May, Daniel Hornburg, Martin H. Schludi, Thomas Arzberger, Kristin Rentzsch, Benjamin M. Schwenk, Friedrich A. Grässer, Kohji Mori, Elisabeth Kremmer, Julia Banzhaf-Strathmann, Matthias Mann, Felix Meissner, Dieter Edbauer

**Affiliations:** 1 German Center for Neurodegenerative Diseases (DZNE), Munich, Schillerstr. 44, 80336 Munich, Germany; 2 Max Planck Institute of Biochemistry, Martinsried, Germany; 3 Center for Neuropathology and Prion Research, Ludwig-Maximilians-University Munich, Feodor-Lynen-Str. 23, 81377 Munich, Germany; 4 Department of Psychiatry and Psychotherapy, Ludwig-Maximilians University Munich, Nußbaumstraße 7, 80336 Munich, Germany; 5 Institute of Virology, Saarland University Medical School, 66421 Homburg, Germany; 6 Adolf Butenandt Institute, Biochemistry, Ludwig-Maximilians University Munich, Schillerstr. 44, 80336 Munich, Germany; 7 Institute of Molecular Immunology, Helmholtz Zentrum München, German Research Center for Environmental Health (GmbH), Marchioninistr. 25, 81377 Munich, Germany; 8 Munich Cluster of Systems Neurology (SyNergy), Munich, Germany

**Keywords:** Neurodegeneration, C9orf72, FTLD, ALS, Unc119, Proteomics

## Abstract

Hexanucleotide repeat expansion in *C9orf72* is the most common pathogenic mutation in patients with amyotrophic lateral sclerosis (ALS) and frontotemporal lobar degeneration (FTLD). Despite the lack of an ATG start codon, the repeat expansion is translated in all reading frames into dipeptide repeat (DPR) proteins, which form insoluble, ubiquitinated, p62-positive aggregates that are most abundant in the cerebral cortex and cerebellum. To specifically analyze DPR toxicity and aggregation, we expressed DPR proteins from synthetic genes containing a start codon but lacking extensive GGGGCC repeats. Poly-Gly-Ala (GA) formed p62-positive cytoplasmic aggregates, inhibited dendritic arborization and induced apoptosis in primary neurons. Quantitative mass spectrometry analysis to identify poly-GA co-aggregating proteins revealed a significant enrichment of proteins of the ubiquitin–proteasome system. Among the other interacting proteins, we identified the transport factor Unc119, which has been previously linked to neuromuscular and axonal function, as a poly-GA co-aggregating protein. Strikingly, the levels of soluble Unc119 are strongly reduced upon poly-GA expression in neurons, suggesting a loss of function mechanism. Similar to poly-GA expression, Unc119 knockdown inhibits dendritic branching and causes neurotoxicity. Unc119 overexpression partially rescues poly-GA toxicity suggesting that poly-GA expression causes Unc119 loss of function. In *C9orf72* patients, Unc119 is detectable in 9.5 % of GA inclusions in the frontal cortex, but only in 1.6 % of GA inclusions in the cerebellum, an area largely spared of neurodegeneration. A fraction of neurons with Unc119 inclusions shows loss of cytosolic staining. Poly-GA-induced Unc119 loss of function may thereby contribute to selective vulnerability of neurons with DPR protein inclusions in the pathogenesis of *C9orf72* FTLD/ALS.

**Electronic supplementary material:**

The online version of this article (doi:10.1007/s00401-014-1329-4) contains supplementary material, which is available to authorized users.

## Introduction

Amyotrophic lateral sclerosis (ALS) and frontotemporal lobar degeneration (FTLD) are severe neurodegenerative diseases with no effective treatment. Degeneration of the upper and lower motor neurons in ALS leads to progressive paralysis [42]. Depending on the affected regions, FTLD patients suffer from dementia, behavioral abnormalities, language impairment and personality changes [21]. Both diseases have overlapping clinical, neuropathological and genetic features and are often described as extreme ends of a disease spectrum [22].

Recently, a mutation in the non-coding region of the *C9orf72* gene has been identified as the most common genetic cause of both ALS and FTLD [12, 20, 41]. Mutation carriers have a GGGGCC hexanucleotide repeat expansion either in the first intron or the promoter region, depending on the isoform of the *C9orf72* transcript [5]. Patients typically have several hundred or thousand repeats, whereas healthy controls show <33 repeats [5, 51]. *C9orf72* patients exhibit clinical symptoms similar to other FTLD or ALS subtypes, but suffer from an unusually high incidence of psychosis [13].

In addition to the common TDP-43 aggregates in FTLD and ALS, *C9orf72* mutation carriers have abundant star-shaped, TDP-43-negative neuronal cytoplasmic inclusions (NCI) particularly in the cerebellum, hippocampus and frontal neocortex that stain positive for markers of the proteasome system (UPS) such as p62 or ubiquitin [1, 7]. We and others discovered that these TDP-43-negative inclusions contain dipeptide repeat proteins (DPR) that are translated ATG-independent from both sense and antisense transcripts of the *C9orf72* repeat in all reading frames [4, 19, 33, 35, 36, 55]. Repeat translation results in five DPR species, poly-GA, poly-GR, poly-GP, poly-PR and poly-PA. Nearly all TDP-43-negative inclusions contain poly-GA, while the other DPR species co-aggregate to a lesser extent. The translation of the DPR proteins is initiated without an ATG start codon, a phenomenon that was initially discovered in other repeat expansion disorders such as myotonic dystrophy 1 and spinocerebellar ataxia type 8 and was recently also found in fragile X-associated tremor/ataxia syndrome (FXTAS) [48, 54].

Several possible disease mechanisms are discussed (reviewed in [18, 32]). First, DPR protein aggregates, or their precursors, may be toxic through binding or sequestration of cellular proteins. Second, both sense and antisense repeat transcripts accumulate in nuclear RNA foci and may cause the sequestration of specific RNA-binding proteins, which potentially impairs the physiological function of those proteins [15, 26, 43]. Third, *C9orf72* mRNA expression is downregulated in patients with a hexanucleotide repeat expansion, which may indicate a loss of function pathomechanism [12, 20]. Currently, the physiological function of *C9orf72* and the relative importance of the three proposed disease mechanisms are still unclear.

The investigation of aggregation and toxicity of DPR proteins is essential to further elucidate their role in disease progression. Therefore, we developed a primary neuronal cell culture model to test the toxicity and aggregation properties of poly-GA, the most abundant of the five DPR species in patient brain [35]. Our cell-based model reproduces key disease features, including formation of insoluble poly-GA aggregates and co-aggregation with p62. Strikingly, poly-GA expression caused neurotoxicity, suggesting that our cell culture model is a valuable tool to study DPR proteins in vitro. To elucidate the mechanism of GA-mediated neurotoxicity, we analyzed the proteome composition of poly-GA aggregates in our model using mass spectrometry-based proteomics. Recently, we have developed a label-free workflow which allows multiple quantitative comparisons of cellular systems [9, 28] and enables an unbiased analysis of protein aggregates from primary cells. Using this approach, we identified Unc119 as a potential new disease-relevant protein, which is co-aggregating in DPR protein inclusions of *C9orf72* patients.

## Materials and methods

### Antibodies and reagents

The following antibodies were used: Anti-GFP (mouse N86/8, Neuromab, Davis, CA, USA and rabbit, Clontech, Mountainview, CA, USA), anti-β-actin (Sigma Aldrich, St. Louis, MO, USA), anti-myc (mouse 9E10 and rabbit A-14, Santa Cruz biotechnology, Dallas, TX, USA), anti-p62 for immunoblotting (Cell Signaling, Danvers, MA, USA), anti-p62 for immunofluorescence (MBL, Nagoya, Japan), anti-HA (Sigma Aldrich), anti-V5 (Life technologies, Carlsbad, CA, USA), anti-TDP43 (Sigma Aldrich), anti-phospho-TDP-43 (Ser409/Ser410, rat, clone 1D3) [38], anti-PSD-95 (K28/43, Neuromab), anti-Unc119 (termed Unc119#2, ThermoFisher scientific, Pierce Biotechnology, Rockford, IL, USA), anti-poly-GA [29], anti-PSMC2 (Bethyl laboratories, Montgomery, TX, USA), anti-PSMC4 (Bethyl laboratories), anti-MAP2 (AP-20, Sigma Aldrich), DAPI (Roche Applied Science, Penzberg, Germany). An Unc119 specific antibody (Unc119#1) was raised and affinity purified against full-length human Unc119 fused to GST in rabbits (Eurogentec, Seraing, Belgium) as described previously [36]. For competition experiments, the diluted Unc119 antibodies were preincubated with native or denatured GST or GST-Unc119 (25 µg/ml) for 2 h at 37 °C. For denaturation, the concentrated GST fusion proteins were heated in 1 % SDS 50 mM Tris pH 8.0 at 95 °C for 5 min. We raised a poly-AP specific monoclonal antibody (clone 14E2 of isotype IgG1) by immunizing rats with synthetic AP_10_ peptide as described previously [29].

### DNA constructs and lentivirus production

Synthetic genes for DPR sequences with ATG start codon, reduced GC content and very few remaining GGGGCC repeats were made to order with C-terminal epitope tags (Life technologies, Geneart, Regensburg, Germany). For details and design rational see Fig. S1a. The full sequence information is available in the supplemental methods. Synthetic genes and the original GGGCCG-based poly-GP construct with an ATG start codon were subcloned into pEF6/V5-His vector (Life technologies) or a lentiviral vector driven by human synapsin promoter (FhSynW2). To replace the ATG start codon in the GA_149_-myc construct with a TAG stop codon we cloned annealed oligonucleotides between an SgrAI site at the 5′ end of the open reading frame and the EcoRI site in the vector. As a negative control GFP from pEGFP-N1 (Clontech) was subcloned into pEF6/V5-His and FhSynW2. The GGGGCC repeat constructs without ATG start codon had been described previously [36]. Rat and human Unc119 cDNA was expressed from a lentiviral vector driven by human ubiquitin promoter containing an N-terminal HA-tag (FUW2-HA). We used shRNA targeting rat Unc119 (GAGAGGCACTACTTTCGAA) or a control targeting firefly luciferase (CGTACGCGGAATACTTCGA) driven by the H1 promoter in the vector FUW coexpressing TagRFP both for transfection and transduction. Lentivirus was produced in HEK293FT cells (Life Technologies) as described previously [17]. The Q_102_-GFP construct in pCS2 vector was a gift from B. Schmid [44].

### Cell culture, immunoblotting and immunofluorescence

HEK293FT cells were transfected using Lipofectamine 2000 according to the manufacturer’s instructions. For immunoblotting, cells were harvested in RIPA buffer (137 mM NaCl, 20 mM Tris pH 7.5, 0.1 % SDS, 10 % Glycerol, 1 % Triton X-100, 0.5 % Deoxycholate, 2 mM EDTA) containing protease and phosphatase inhibitor cocktails (Sigma). Cells were lysed on ice for 20 min and centrifuged at low speed to avoid pelleting of the DPR protein aggregates (1,000*g* for 10 min at 4 °C). The supernatant was mixed with 4 × loading buffer (0.4 M sodium phosphate pH 7.5, 8 % SDS, 40 % glycerol, 10 % 2-mercaptoethanol, bromphenol blue) and incubated at 95 °C for 5 min. Primary hippocampal or cortical neurons were cultured from embryonic day 19 rats and infected with lentiviruses as described previously [17, 47]. Primary cortical neurons infected with indicated lentiviruses were harvested with 2x loading buffer. Samples were run on 12.5 % SDS-PAGE gels or Novex 10–20 % Tris-Tricine gels (Life technologies).

HEK293FT cells and primary neurons were fixed for 10 min with 4 % paraformaldehyde and 4 % sucrose. Primary and secondary antibodies were diluted in GDB buffer (0.1 % gelatine, 0.3 % Triton X-100, 450 mM NaCl, 16 mM sodium phosphate pH 7.4). Confocal images were obtained on a confocal laser scanning LSM710 system (Carl Zeiss, Jena) with a 40 × oil immersion objective. Sholl analysis was performed manually and blinded to the experimental conditions using MetaMorph software as described before [47].

### Filter trap assay

To detect DPR aggregates, transfected HEK293FT cells or transduced neurons were harvested with 1 % Triton X-100, 50 mM MgCl_2_ and 0.2 mg/ml DNase I in PBS. After centrifugation (18,000*g* for 30 min at 4 °C) the pellet was resuspended in 2 % SDS in 100 mM Tris (pH 7.0). After 1 h incubation at room temperature the homogenates were filtered through a Whatman cellulose acetate membrane with 0.2 µm pore size (Sigma Aldrich).

To detect Unc119 aggregates, brain samples were resuspended in RIPA buffer containing 0.2 mg/ml DNase I. After centrifugation (186,000*g* for 30 min at 4 °C) the pellet was resuspended in 1 % SDS in 100 mM Tris (pH 7.0) and treated as above.

### Cellular assays

Viability of HEK293FT cells and primary neurons was analyzed according to the manufacturer’s instructions in 96 well plates: LDH Cytotox Non-Radioactive cytotoxicity assay (Promega), Caspase-glo 3/7 assay (Promega), TUNEL in situ cell death detection TMR red assay (Roche). For the TUNEL assay dead and living cells were counted manually with the Fiji cell counter plugin. At least 400 cells per condition were counted per experiment in a total of three independent experiments. Proteasome activity was measured using the Proteasome-Glo kit according to the manufacturer’s instructions (Promega).

### qPCR

RT-qPCR of primary cortical neurons was performed as described previously [39]. The following primers were used for analysis of rat Unc119: GCGCTTTGTTCGATACCAGT and TGTTCTTGCTGCTGGGAATG. GAPDH was used as a reference gene: CCGCATCTTCTTGTGCAGTGCC and AGACTCCACGACATACTCAGCACC.

### Immunoprecipitation of poly-GA aggregates

Transduced cortical neurons or transfected HEK293FT cells were harvested in RIPA buffer as described above, additionally adding Benzonase (67 U/ml). Samples were rotated for 30 min at 4 °C prior to centrifugation (1,000*g* for 15 min at 4 °C). 2 % of the input was kept and the rest of the supernatant was added to 50 µl protein G dynabeads (Life Technologies), that were preincubated with 10 µg GFP antibody. After incubation (3 h at 4 °C) the magnetic beads were washed three times (150 mM NaCl, 50 mM Tris pH 7.5, 5 % Glycerol). One-fifth of the bead-mix was denatured in 4× loading buffer (95 °C, 5 min) for western blot analysis and the rest was kept for mass spectrometry (MS) analysis. For co-immunoprecipitations from transfected HEK293FT cells the whole samples were analyzed by western blot.

### Sample preparation for MS

The bead-mix was resuspended in 50 µl 8 M Urea, 10 mM Hepes pH 8.0. Protein cysteines were reduced with DTT and alkylated with iodoacetamide (IAA), followed by quenching of IAA with thiourea. Proteins were digested with LysC for 4 h and the bead-mix was centrifuged for 5 min at 16,000*g*. The supernatant was removed and diluted with 4 volumes of 50 mM ammonium bicarbonate. The pellet was resuspended in 1 volume 6 M urea, 2 M thiourea, 10 mM Hepes pH 8.0, 4 volumes 50 mM ammonium bicarbonate and LysC. Trypsin was added to both fractions and the final digest was carried out for 16 h. The resulting peptide mix was desalted on C18 StageTips [40] and analyzed in single shots. Notably, in the supernatant we quantified only 50 proteins (data not shown) whereas over-night digestion of the pellet with LysC and trypsin resulted in over 450 quantifications.

### LC–MS/MS

Peptides were separated on a Thermo Scientific EASY-nLC 1000 HPLC system (Thermo Fisher Scientific, Odense, Denmark) via in-house packed columns (75 μm inner diameter, 20 cm length, 1.9 μm C18 particles (Dr. Maisch GmbH, Germany)) in a 100 min gradient from 2 % acetonitrile, 0.5 % formic acid to 80 % acetonitrile, 0.5 % formic acid at 400 nl/min. The column temperature was set to 50 °C. An Orbitrap mass spectrometer [34] (Orbitrap Elite, Thermo Fisher Scientific) was directly coupled to the LC via nano electrospray source. The Orbitrap Elite was operated in a data-dependent mode. The survey scan range was set from 300 to 1,650 m/z, with a resolution of 120,000. Up to the five most abundant isotope patterns with a charge ≥2 were subjected to collision-induced dissociation fragmentation at a normalized collision energy of 35, an isolation window of 2 Th and a resolution of 15,000 at m/z 200. Data was acquired using the Xcalibur software (Thermo Scientific).

### MS data analysis and statistics

To process MS raw files, we employed the MaxQuant software (v 1.4.0.4) [9] and Andromeda search engine [11], against the UniProtKB Rat FASTA database (06/2012) using default settings. Enzyme specificity was set to trypsin allowing cleavage N-terminally to proline and up to 2 miscleavages. Carbamidomethylation was set as fixed modification, acetylation (N-terminus) and methionine oxidation were set as variable modifications. A false discovery rate (FDR) cutoff of 1 % was applied at the peptide and protein level. ‘Match between runs’, which allows the transfer of peptide identifications in the absence of sequencing, was enabled with a maximum retention time window of 1 min. Protein identification required at least one razor peptide. Data were filtered for common contaminants (*n* = 247). Peptides only identified by site modification were excluded from further analysis. A minimum of two valid quantifications was required in either GA_149_-GFP or GFP quadruplicates.

For bioinformatic analysis as well as visualization, we used the open PERSEUS environment, which is part of MaxQuant and the R framework (Team, R Development Core, 2008). Imputation of missing values was performed with a normal distribution (width = 0.3; shift = 1.8). For pairwise comparison of proteomes and determination of significant differences in protein abundances, *t* test statistics were applied with a permutation-based FDR of 2 % and S0 of 2 [50]. For GA-aggregate interacting proteins 1D annotation enrichment on the Welch-test difference using Uniprot Keywords with a Benjamini–Hochberg corrected FDR of 2 % showed a significant enrichment of the annotations for the ubiquitin–proteasome system (gene ontology molecular function: “ubiquitin binding”, pfam: “ubiquitin”, uniprot keywords: “proteasome”) (*p* value = 8.7^−11^, score = 0.77, 5.7-fold enrichment) [10].

### Patient samples

All patient materials were provided by the Neurobiobank Munich, Ludwig-Maximilians-University (LMU) Munich and were collected and distributed according to the guidelines of the local ethical committee. Clinical data are listed in Table S1. Immunohistochemistry and immunofluorescence stainings were performed as described previously [35]. For competition experiments the Unc119#1 antibody was preincubated with 0.25 µg/µl native GST or GST-Unc119 for 2 h at 37 °C. To compare poly-GA aggregates from patient tissue with aggregates from neuronal culture, non-fixed brain-tissue sample of 1 mm in diameter was smeared between two slides and fixed and stained like cultured neurons. For quantification of Unc119 and GA co-aggregation in the different brain regions three patients were manually analyzed. In each region at least 300 GA aggregates were counted per patient.

## Results

### Poly-GA forms p62-positive SDS-resistant aggregates in HEK293 cells

**Fig. 1:**
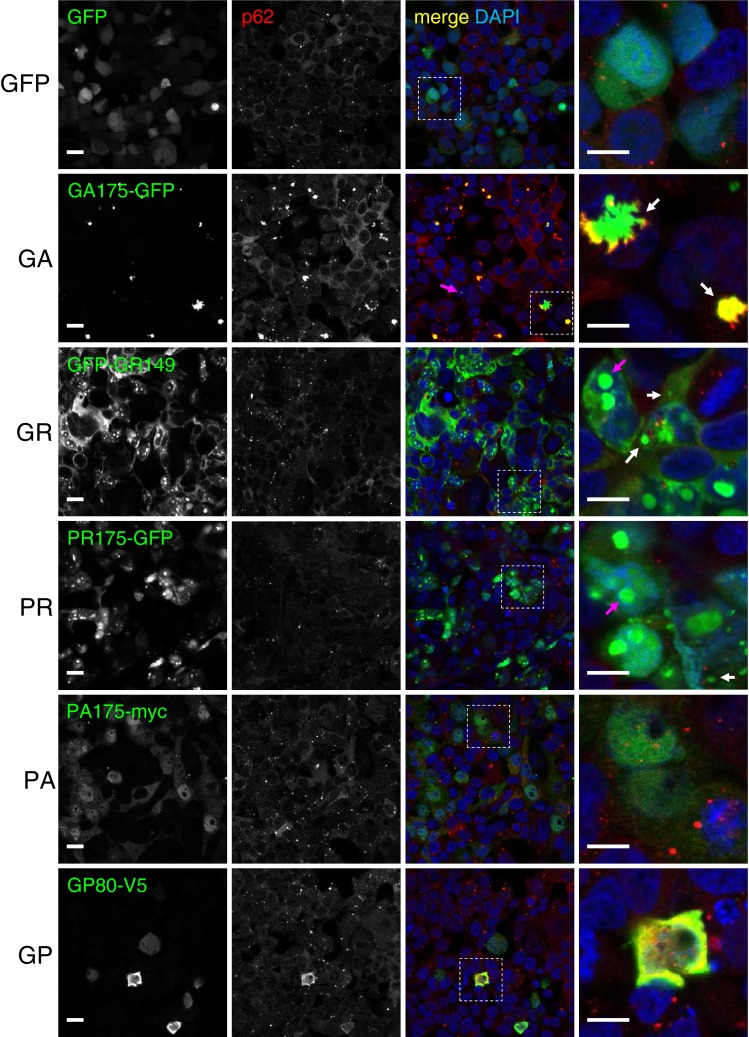
DPR species show differential aggregation properties in HEK293 cells. HEK293 cells were transfected with the five different DPR constructs (GA_175_-GFP, GFP-GR_149_, PR_175_-GFP, PA_175_-myc and GP_80_-V5) or GFP as a control and analyzed 2 days later by GFP fluorescence or in case of PA_175_-myc and GP_80_-V5 by immunofluorescence using specific antibodies. DAPI was used as a nuclear marker. Cytoplasmic inclusions (*white arrows*) and nuclear inclusions (*magenta arrows*) are seen for GA_175_-GFP, GFP-GR_149_ and PR_175_-GFP. Many dot-like and star-shaped GA_175_-GFP inclusions co-localize with p62 (*second column from the left*). *Right panels* show close-ups of areas indicated in the *merge column*. Magnifications of intranuclear GA_175_-GFP inclusions are shown in Fig. S2a. Negative control stainings are shown in Fig. S2b. *Scale bar* represents 15 µm for overview and 5 µm for close-up

To investigate the characteristics of the five different DPR species in cell culture, we generated ATG-initiated epitope-tagged expression constructs for all reading frames of the GGGGCC repeat (Fig. S1a). These synthetic constructs, encoding 149–175 repeats, contain a mixture of alternative codons with reduced GC content to prevent instability observed with repetitive GGGGCC-based constructs in *E. coli,* while allowing for high expression in mammalian cells (Fig. 1). Moreover, changing the original hexanucleotide repeat sequence, but maintaining the DPR protein sequence, allowed us to focus on protein toxicity rather than GGGGCC or CCCCGG RNA toxicity. Unfortunately, gene synthesis for poly-GP constructs repeatedly failed. Thus, we generated an ATG-initiated construct from the endogenous repeat sequence encoding about 80 GP repeats. Importantly, without an ATG start codon GGGGCC repeat constructs did not impair cell viability in HEK293 cells excluding overt RNA toxicity of the utilized constructs (Fig. S1b).

To verify protein expression, we transfected HEK293 cells with the DPR constructs (Fig. S1c). We observed protein products of the expected size for GP_80_-V5 (21 kDa), but not for GA_175_-GFP (50 kDa), GFP-GR_149_ (68 kDa), PR_175_-GFP (71 kDa) and PA_175_-myc (31 kDa). Similar to immunoblots from patient brains [36], specific bands at the top of the gel for GA_175_-GFP, GFP-GR_149_, PR_175_-GFP and PA_175_-myc indicate formation of insoluble aggregates for these species (Fig. S1c).

To compare the localization and aggregation of the different DPR species we analyzed transfected HEK293 cells by immunofluorescence (Fig. 1, S2a/b). Strikingly, poly-GA, the most abundant DPR species in patients [35, 36], predominantly formed distinct dot-like or star-shaped inclusions in the cytosol (Fig. 1) and occasionally in the nucleus (Fig. S2a). In contrast, GFP-GR_149_ showed mainly cytoplasmic staining. PR_175_-GFP was diffusely localized, both in the cytosol and nucleus. Additionally, GFP-GR_149_ and PR_175_-GFP expressing cells often showed large dot-like intranuclear inclusions and occasionally smaller cytoplasmic inclusions. In contrast, poly-PA was evenly distributed throughout the nucleus and cytoplasm without apparent aggregation. GP_80_-V5 was distributed throughout the cytoplasm without forming compact inclusions (Fig. 1).

The poly-GA inclusions were strongly positive for p62 (Fig. 1), suggesting that cytoplasmic poly-GA forms ubiquitinated aggregates similar to the abundant poly-GA inclusions found in *C9orf72* FTLD/ALS [36]. Some GP_80_-V5 expressing cells also showed increased p62 levels and co-localization with GP_80_-V5. For the other DPR species no such co-localization was detected. Moreover, immunofluorescence and immunoblotting showed overall increased p62 levels only in GA_175_-GFP expressing cells (Fig. 1, S1c). In HEK293 none of the constructs induced cell death in an LDH release assay (Fig. S2c).

To confirm aggregation of the DPR proteins, we performed a filter trap assay with HEK293 extracts in the presence of 2 % SDS. Insoluble GA_175_-GFP and GFP-GR_149_ aggregates were readily detectable on the cellulose acetate filter even upon 125-fold dilution, but no signal was detected for GP_80_-V5, PR_175_-GFP and AP_175_-myc with specific antibodies under these conditions suggesting that they are less aggregation prone and can be solubilized at 2 % SDS in the filter trap assay, but not at 0.1 % SDS in polyacrylamide gels (compare Fig. S1c and S2d).

Taken together, GA_175_-GFP, GFP-GR _149_ and PR_175_-GFP DPR proteins formed cytoplasmic or nuclear inclusions in HEK293 cells. Although the number of repeats was different for the individual constructs, these data suggest differential solubility of the five DPR species, since AP_175_-myc, one of the longest constructs, apparently, remained soluble under these conditions even when omitting the GFP tag. However, we cannot exclude that longer repeats (on average 1,000–2,000) observed in patients may promote aggregation of all DPR species. Importantly, poly-GA mimicked most closely the pathology in patient brain by forming compact p62-positive cytoplasmic inclusions and SDS-resistant aggregates and was therefore used for all subsequent experiments.

### Poly-GA forms inclusion in primary hippocampal and cortical neurons

Poly-GA expression in HEK293 cells recapitulates all known features of DPR inclusions seen in *C9orf72* patients, without causing toxicity (Fig. S2c). However, DPR proteins are almost exclusively expressed in neurons [4, 36] and the *C9orf72* mutation leads to selective degeneration of neurons. Thus, we analyzed the effects of long-term expression of poly-GA in post-mitotic neurons using lentiviral transduction.

**Fig. 2:**
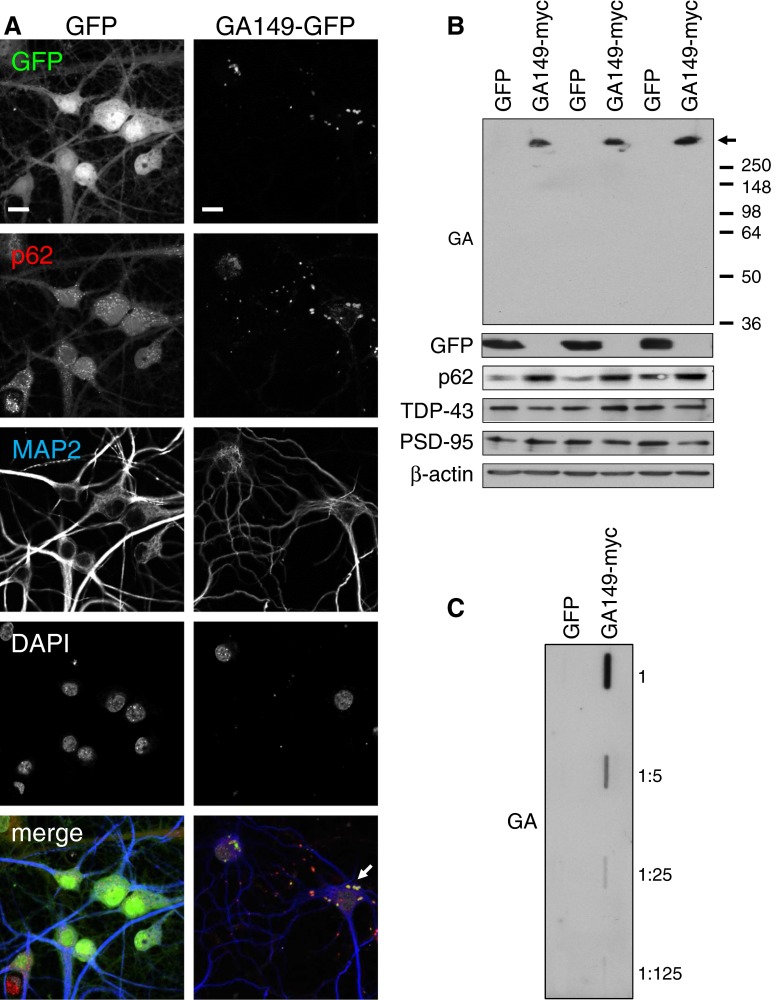
Poly-GA forms p62-positive aggregates in neurons. **a** Immunofluorescence of primary hippocampal neurons transduced with GA_149_-GFP or GFP control lentivirus at day 6 in vitro for 15 days (DIV6 + 15). Immunostaining for p62 and the dendritic marker protein MAP2. DAPI was used a nuclear marker. Poly-GA forms p62-positive inclusions (*arrow*) in the soma and dendrites. *Scale bar* 15 µm. **b** Immunoblotting of primary cortical neurons transduced with GA_149_-myc or GFP control lentivirus (DIV6 + 17) with the indicated antibodies. Poly-GA aggregates are stuck at the top of the gel (*arrow*). GA_149_-myc induces upregulation of p62, but levels of TDP-43 and the synaptic marker protein PSD-95 are not affected. Three separate transductions are shown. **c** Filter trap assay of primary cortical neurons transduced with GA_149_-myc or GFP (DIV6 + 17). Poly-GA aggregates are detected in the serial dilution of homogenates using anti-GA

Lentiviral expression of GA_149_-GFP in primary rat hippocampal neuron cultures resulted in compact p62-positive poly-GA inclusions (Fig. 2a) similar to the results in HEK293 cells (Fig. 1) and patients [36]. Poly-GA/p62-positive dot-like structures were most common in the cell soma, but were also detectable within dendrites. This finding is reminiscent of the poly-GA-positive dystrophic neurites seen in patient brains [29, 36]. Importantly, the DPR inclusions in transduced neurons and patient neurons showed comparable poly-GA staining intensities suggesting that ATG-driven expression in neurons is a valid model to study DPR toxicity in vitro (Fig. S3). In immunoblots of neuronal extracts all poly-GA protein was retained at the top of the gel indicative of high molecular weight aggregates (Fig. 2b). Consistent with the data in HEK293 cells (Fig. 1, S1c) and patient data [2, 49], p62 levels were strongly increased in poly-GA expressing cells. In contrast, TDP-43 levels were unaffected by GA_149_-myc expression (Fig. 2b) and pathological TDP-43 phosphorylation could not be detected (data not shown). Filter trap analysis further corroborated the formation of SDS-resistant poly-GA aggregates in primary neurons (Fig. 2c).

### Poly GA is toxic in primary hippocampal and cortical neurons

**Fig. 3:**
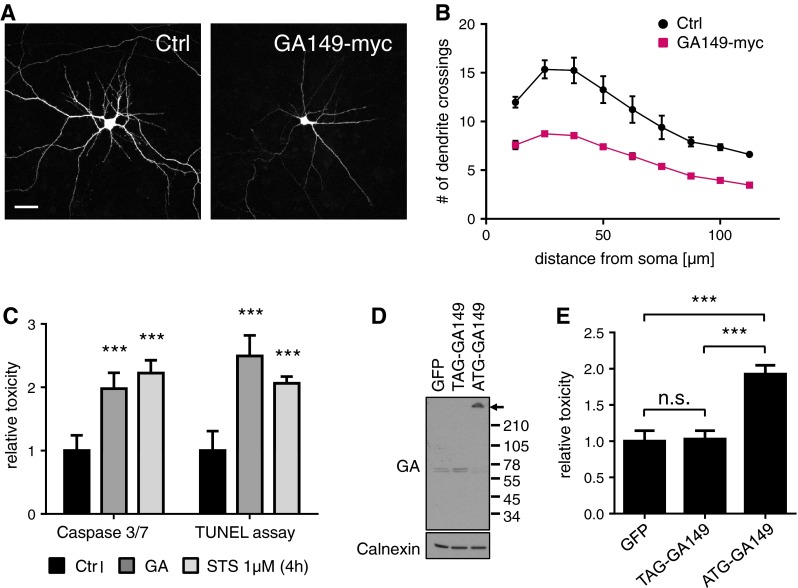
Poly-GA causes dendrite loss and induces apoptosis in primary neurons. **a** Cortical neurons were co-transfected with empty vector as control (Ctrl) or GA_149_-myc together with GFP to outline cell morphology (DIV7 + 4). *Scale bars* represent 40 µm. **b** Dendritic complexity was measured using Sholl analysis by manually counting the number of dendrites crossing concentric circles around the soma. Poly-GA expression leads to significant reduction of dendritic branching. *N* = 3 with 40 cells analyzed per condition in each experiment, mean ± SEM. *p* < 0.001 for 12.5 µm radius, *p* < 0.0001 from 25 to 50 µm radius, *p* < 0.001 for 62.5 µm radius, *p* < 0.01 for 75 µm radius and *p* < 0.05 from 87.5 to 112.5 µm radius (two-way ANOVA). **c** Apoptosis in transduced neurons was analyzed using a fluorogenic assay to detect caspase 3/7 activation and a TUNEL assay to detect apoptotic DNA fragmentation (DIV6 + 17). Caspase 3/7 activity was increased 2.0-fold in GA_149_-myc transduced cortical neurons. TUNEL-positive apoptotic cells (manually counted using the Fiji cell count plug-in) were increased by 2.5-fold in GA_149_-myc transduced hippocampal neurons compared to control cells. Representative images of TUNEL stainings are shown in Fig. S2. DIV6 + 17. n = 3 experiments with 6 replicates each; mean ± SD, Student’s *t* test, ***p < 0.001. **d** Immunoblots of cortical neurons transduced with GA_149_-myc constructs with or without start codon (DIV8 + 10). Replacing the ATG start codon in the synthetic GA_149_-myc gene with a TAG stop codon prevents poly-GA expression and aggregation. *Arrow* indicates top of the gel. **e** LDH release assay detected neurotoxicity of GA_149_-myc only in the presence of an ATG start codon in transduced cortical neurons (DIV8 + 14). One-way ANOVA with Tukey’s post-test. ***p < 0.001, *n* = 3 with six replicates in each experiment

Whether DPR proteins contribute to neurodegeneration in *C9orf72* patients is still unclear. In GA_149_-GFP expressing cultures, the neuron density appeared lower although the remaining cells maintained the typical neuronal morphology. However, neurite branching as judged by MAP2 staining appeared less complex (Fig. 2a). Therefore, we quantified dendritic complexity by Sholl analysis, which confirmed reduced branching in GA_149_-myc transfected neurons (Fig. 3a, b).

Furthermore, we quantified neuronal apoptosis in lentivirus transduced cells using several different methods. Compared to controls, GA_149_-myc expressing cortical neurons showed a highly significant 2.0-fold increase in Caspase 3/7 activity (Fig. 3c). Moreover, by analyzing apoptotic DNA fragmentation in primary hippocampal neurons using TUNEL labeling, we detected a highly significant 2.5-fold increase in the number of apoptotic cells (Fig. 3c, compare Fig. S4). Neurotoxicity was also associated with enhanced LDH release in GA_149_-myc expressing cells (Fig. 3e).

To exclude that the synthetic non-GGGGCC repeat sequence encoding GA_149_-myc in our constructs causes RNA-mediated toxicity we replace the ATG start codon with a stop codon (TAG-GA_149_-myc, compare Fig. S1a). Without a start codon we detected no poly-GA expression from the synthetic GA_149_-myc gene upon transduction of primary neurons (Fig. 3d) indicating that this non-GGGGCC construct does not support RAN translation. Importantly, TAG-GA_149_-myc did not impair viability suggesting that the ATG-GA_149_-myc construct causes neurotoxicity due to poly-GA expression and not due to RNA toxicity (Fig. 3e). Therefore, ATG-driven poly-GA expression constructs were used for the remainder of this study.

In summary, poly-GA formed p62-positive inclusions as seen in neurons of patients with *C9orf72* mutation and induced apoptosis in primary cortical and hippocampal neurons, suggesting an important role of poly-GA in the pathogenesis of *C9orf72* FTLD/ALS.

### Poly-GA co-aggregates with components of the ubiquitin–proteasome system and the cargo adaptor Unc119

**Fig. 4:**
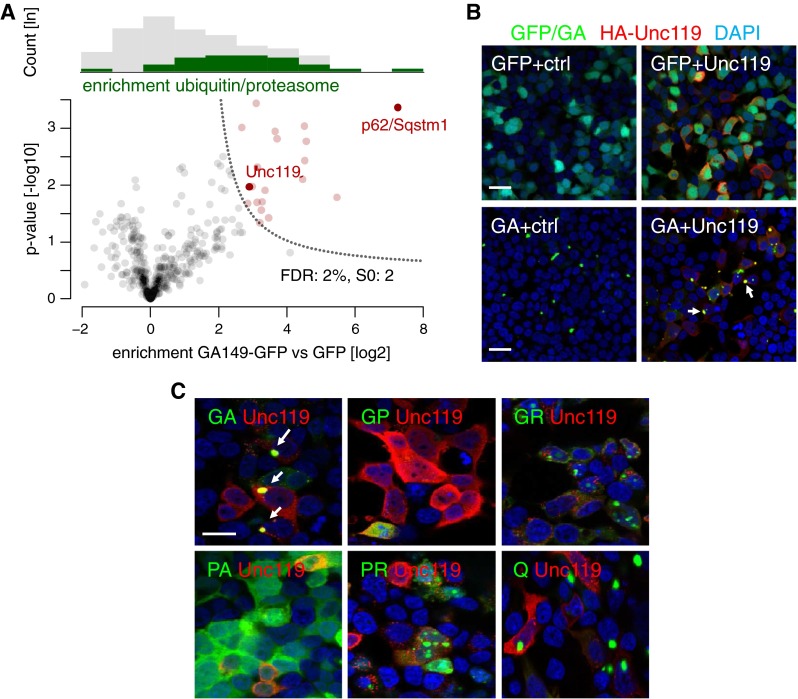
Unc119 specifically co-aggregates with poly-GA. **a** Quantitative proteomics of GFP immunoprecipitations from primary cortical neurons transduced with GFP or GA_149_-GFP (DIV6 + 17). p62/Sqstm1 shows highest enrichment and statistical significance. Unc119 was identified by two unique peptides (GGGGTGPGAEPVPGASNR and LGPLQGK) and one peptide (YQFTPAFLR) shared with its homolog Unc119b. Full protein names are listed in Table 1. *Upper panel* illustrates distribution of quantified protein abundances binned for enrichment factors (*x*-axis below). Enrichment of ubiquitin-related and proteasomal proteins in the poly-GA interactome is highlighted in green. *Lower panel* depicts volcano plot showing poly-GA interacting proteins. False discovery rate (FDR) controlled statistical analysis identified 20 poly-GA interacting proteins compared to control (*red dots*). *Dotted line* depicts threshold for statistical significance. **b** Immunofluorescence of HEK293 cells co-transfected with GFP or GA_175_-GFP and HA-Unc119 or empty vector control (ctrl). Staining with HA and GFP antibodies and DAPI as nuclear marker. Many GA_175_-GFP inclusions show co-aggregation of HA-Unc119 (examples marked with *arrows*). Separate channels of these images are shown in Fig. S6. *Scale bar* 30 µm. **c** HEK293 cells were transfected with the five different poly-DPR constructs (GA_175_-GFP, GFP-GR_149_, PR_175_-GFP, PA_175_-myc and GP_80_-V5) or Q_102_-GFP and analyzed using GFP fluorescence and immunostaining of HA-Unc119, PA_175_-myc and GP_80_-V5 using specific antibodies 2 days later. HA-Unc119 co-aggregates only with GA_175_-GFP (*white arrows*). DAPI (*in blue*) was used as a nuclear marker. *Scale bar* 20 µm

Since DPRs are highly unusual proteins, we wondered if DPR inclusions sequester endogenous proteins and could thereby contribute to disease progression. To this end, we transduced primary cortical neurons with a lentivirus expressing GA_149_-GFP or GFP alone and immunoprecipitated the interacting proteins with anti-GFP in quadruplicates (Fig. S5a). To identify co-aggregating proteins by an unbiased approach we applied label-free quantitative proteomics. By comparing relative protein abundances in GA_149_-GFP and GFP samples we quantified 450 proteins, 20 of which were strongly enriched in poly-GA aggregates (Fig. 4a, Table 1).

**Table 1:** Poly-GA interacting proteins identified by quantitative MS analysis

Gene names	Protein IDs	−log_10_(p)	log_2_(GA/ctrl)	Protein names
Sqstm1	O08623-2; O08623; O08623-3	3.37	7.25	p62/Sequestosome-1
Rad23a; Rad23b	Q4KMA2; Q5XFX7	1.79	5.47	UV excision repair protein RAD23 homolog A and B
Ubqln2; Ubqln4	D4AA63; D4A3P1	2.77	4.57	Ubiquilin 2; Ubiquilin 4
Sdcbp	Q9JI92	2.43	4.52	Syntenin-1
Ubb; Ubc; Uba52; Rps27a; LOC100360645	G3V9Z2; P0CG51; Q63429; F1LML2; Q6P7R7; P62986; Q6PED0; P62982; F1M516	3.04	4.51	Polyubiquitin-B; Ubiquitin; Polyubiquitin-C; Ubiquitin; Ubiquitin-related; Ubiquitin-60S ribosomal protein L40; Ubiquitin; 60S ribosomal protein L40; Ubiquitin-40S ribosomal protein S27a; Ubiquitin; 40S ribosomal protein S27a
Klhdc10	Q5U3Y0; *D3ZUK9*	2.10	4.46	Kelch domain-containing protein 10
Bag6	Q6MG49; Q6MG49-2	2.81	3.71	Large proline-rich protein BAG6
Psmb6	P28073	2.95	3.65	Proteasome subunit beta type-6
Ubqln1	*F1M971*; Q9JJP9	1.42	3.47	Ubiquilin 1
Dbn1	Q07266; Q07266-2; C6L8E0	1.91	3.36	Drebrin
Myh10; Myh14	Q9JLT0; G3V9Y1; F1LNF0	1.70	3.28	Myosin-10; Myosin-14
Efhd1; Efhd2	Q4FZY0; D4A9T5	1.56	3.25	EF-hand domain-containing protein D1; EF-hand domain-containing protein D1
Acat2	Q5XI22; F1LS48	1.34	3.21	Acetyl-CoA acetyltransferase, cytosolic
Mlf2	D3ZPN3	2.32	3.13	Myeloid leukemia factor 2
Psmc6	G3V6W6	1.70	3.12	Proteasome (prosome, macropain) 26S subunit, ATPase, 6
Psmb4	*D4A640*; P34067; G3V8U9	3.44	3.10	Proteasome subunit beta type-4
Psmb5	G3V7Q6; P28075	1.97	2.98	proteasome (prosome, macropain) subunit, beta type, 5
Unc119; Unc119b	Q62885; *F1LZN2*; D3ZY58	1.97	2.90	Protein unc-119 homolog A and B
Myo5b; Myo5c	F1M111; F1M3R4; P70569	1.68	2.85	Myosin VB/C
Psmd13	B0BN93	3.01	2.68	26S proteasome non-ATPase regulatory subunit 13

Protein enrichment in GA_149_-GFP immunoprecipitates compared to GFP control determined by quantitative mass spectrometry. Listed are the NCBI gene names, the UniProt identifier, logarithmic *p* value and enrichment factor. Statistical analysis using *t* test at a false discovery rate of 2 % and S0 of 2 [50]. Proteins with shared peptides are clustered in groups. Graphical representation is depicted in Fig. 4a

Importantly, p62/Sqstm1, a marker protein for DPR inclusions [4, 33, 36, 55], showed strongest enrichment (Fig. 4a), which is consistent with p62 upregulation (Fig. 2b) and p62/GA co-localization (Fig. 2a). Proteasomal subunits (e.g., PSMB6) and other ubiquitin-related proteins (e.g., Ubiquilin 1 and 2) were 5.7-fold enriched in the poly-GA interactome (*p* value = 8.7 × 10^−11^) (Fig. 4a; Table 1). However, chymotrypsin-like, trypsin-like and caspase-like protease activities associated with the proteasome was not impaired in HEK293 cells expressing poly-GA (Fig. S5b). Moreover, the levels of two proteasomal proteins, PSMC2 and PSCM4, were unaffected by poly-GA expression in HEK293 cells and neurons (Fig. S5c/d). TDP-43 was not identified as poly-GA co-aggregating protein which is in line with the lack of significant co-localization in patients [4, 33, 35, 36, 55]. Interestingly, one of the interaction partners, Unc119, which was 7.5-fold enriched in the GA_149_-GFP immunoprecipitates, was previously identified through severely impaired locomotion in a *C. elegans* mutant and is required for axon development and maintenance [23, 30], which warranted further analysis in the context of ALS. Moreover, Unc119 binds to a myristoylated GAGASA motif of Transducin α (GNAT1), which bears strong resemblance to poly-GA [53]. To confirm that Unc119 interacts and co-aggregates with poly-GA, we co-expressed HA-tagged Unc119 with GA_175_-GFP in HEK293 cells. This resulted in pronounced co-localization of HA-Unc119 with GA_175_-GFP inclusions, which is in contrast to the diffuse cytoplasmic localization of HA-Unc119 in GFP expressing cells (Fig. 4b, S6). Co-immunoprecipitation of HA-Unc119 with both GA_175_-GFP and GA_149_-myc, but not GFP attests that the interaction is indeed mediated by poly-GA (Fig. S5e). In addition, upon co-expression in HEK293 cells, Unc119 did not co-aggregate with the other DPR species (GFP-GR_149_, PR_175_-GFP, GP_80_-V5 and PA_175_-myc) or Q_102_-GFP, an unrelated aggregating protein [44], supporting a specific interaction of Unc119 and poly-GA (Fig. 4c).

**Fig. 5:**
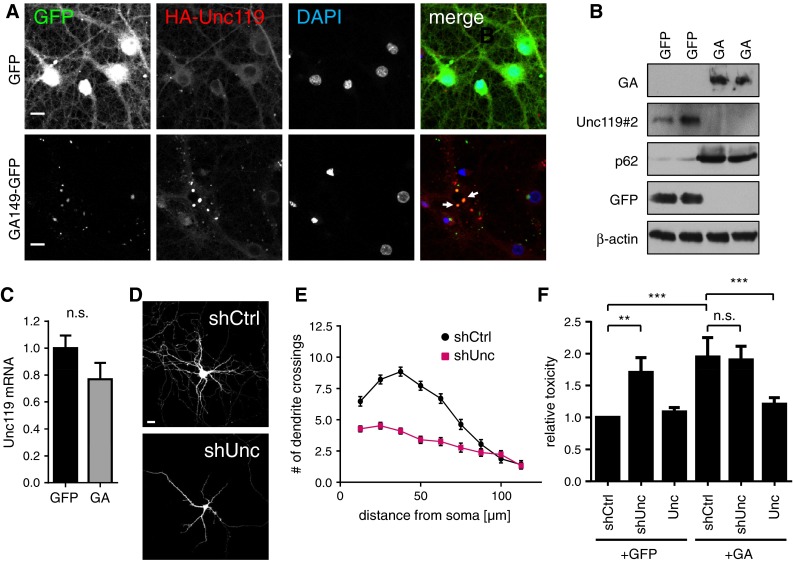
Unc119 sequestration in neurons contributes to poly-GA toxicity. **a** Immunofluorescence of primary hippocampal neurons co-transduced with HA-Unc119 and either GFP or GA_149_-GFP (DIV6 + 17). *Arrows* indicate examples of poly-GA inclusions showing co-aggregation of HA-Unc119. *Scale bar* 15 µm. **b** Immunoblot with the indicated antibodies in GFP or GA_149_-myc transduced cortical neurons shows decreased levels of soluble Unc119 running at 27 kDa. Two separate transductions are shown (DIV6 + 17). **c** qPCR analysis of neurons transduced as in (**b**) shows no significant changes in Unc119 mRNA levels (mean ± SD, Student’s *t* test, DIV7 + 10). (**d**, **e**) Hippocampal neurons transfected with shRNA targeting Unc119 (shUnc) or a non-targeting control (shCtrl) together with GFP to outline cell morphology (DIV7 + 5). Dendritic branching was quantified by Sholl analysis. Unc119 knockdown reduced dendrite complexity significantly (*p* < 0.0001 for 12.5–62.5 µm radius and *p* < 0.001 for 75 µm radius, two-way ANOVA, *n* = 40 neurons per condition). *Scale bar* depicts 40 µm. **f** LDH release assay from cortical neurons co-transduced with either GFP or GA_149_-GFP (GA) together with HA-Unc119 (Unc), shRNA targeting Unc119 (shUnc) or non-targeting shRNA (shCtrl) (DIV6 + 17). Note that Unc119 knockdown causes toxicity in GFP-transduced neurons, but does not increase poly-GA toxicity further. HA-Unc119 expression rescues GA_149_-GFP toxicity. One-way ANOVA with Tukey’s post-test. **p < 0.01, ***p < 0.001, *n* = 3 with six replicates in each experiment

Lentiviral co-expression of Unc119 with GA_149_-GFP in hippocampal neurons further corroborated the specific co-aggregation of Unc119 with poly-GA (Fig. 5a). Neurons with poly-GA aggregates showed bright Unc119 inclusions, suggesting that a large fraction of cellular Unc119 becomes sequestered in poly-GA inclusions.

In summary, identification of the poly-GA interactome provides proteomic evidence for involvement of the ubiquitin–proteasome system and suggests additional molecular targets of poly-GA toxicity through co-aggregation or sequestration.

### Unc119 sequestration contributes to poly-GA toxicity

To analyze how poly-GA inclusions affect endogenous Unc119 we raised a polyclonal antibody against full-length human Unc119 (termed Unc119#1) and tested a commercially available antibody (termed Unc119#2). Both antibodies detected overexpressed rat and human Unc119 (Fig. S7a). To validate both antibodies on endogenous protein, we used RNAi to knockdown Unc119. Lentiviral expression of an Unc119 specific shRNA in neurons strongly reduced Unc119 mRNA levels compared to control cells (Fig. S7b). Both Unc119 antibodies detected robust knockdown of endogenous Unc119 protein by immunoblotting and immunofluorescence, thus confirming their specificity (Fig. S7c–e).

Although Unc119 was enriched in the poly-GA immunoprecipitation (Fig. 4a), almost no Unc119 could be detected at the regular size (27 kDa) in extracts of GA_149_-myc expressing neurons by immunoblotting compared to the GFP expressing control (Fig. 5b). Since the Unc119 mRNA levels remained unchanged (Fig. 5c), this indicates that Unc119 sequestered in poly-GA aggregates becomes insoluble.

To analyze the effect of Unc119 loss of function in neurons we transfected hippocampal neurons with specific shRNAs and analyzed neuron morphology. Unc119 knockdown led to dendrite withering similar to poly-GA expression (Fig. 5d, e). Moreover, compared to a control shRNA, lentiviral Unc119 knockdown induced neuronal death as quantified by increased LDH release (Fig. 5f). While overexpression of HA-Unc119 alone had no effect on cell viability, HA-Unc119 overexpression reduced toxicity in GA_149_-myc expressing neurons suggesting that Unc119 loss of function contributes to poly-GA toxicity in neurons. In contrast, Unc119 knockdown in GA_149_-myc expressing neurons did not increase toxicity, which also indicates that Unc119 loss of function is a major source of poly-GA toxicity (Fig. 5f).

In summary, sequestration of Unc119 in poly-GA aggregates may cause Unc119 loss of function and contribute to FTLD/ALS pathogenesis.

### Unc119 is a component of DPR inclusions in *C9orf72* patients

**Fig. 6:**
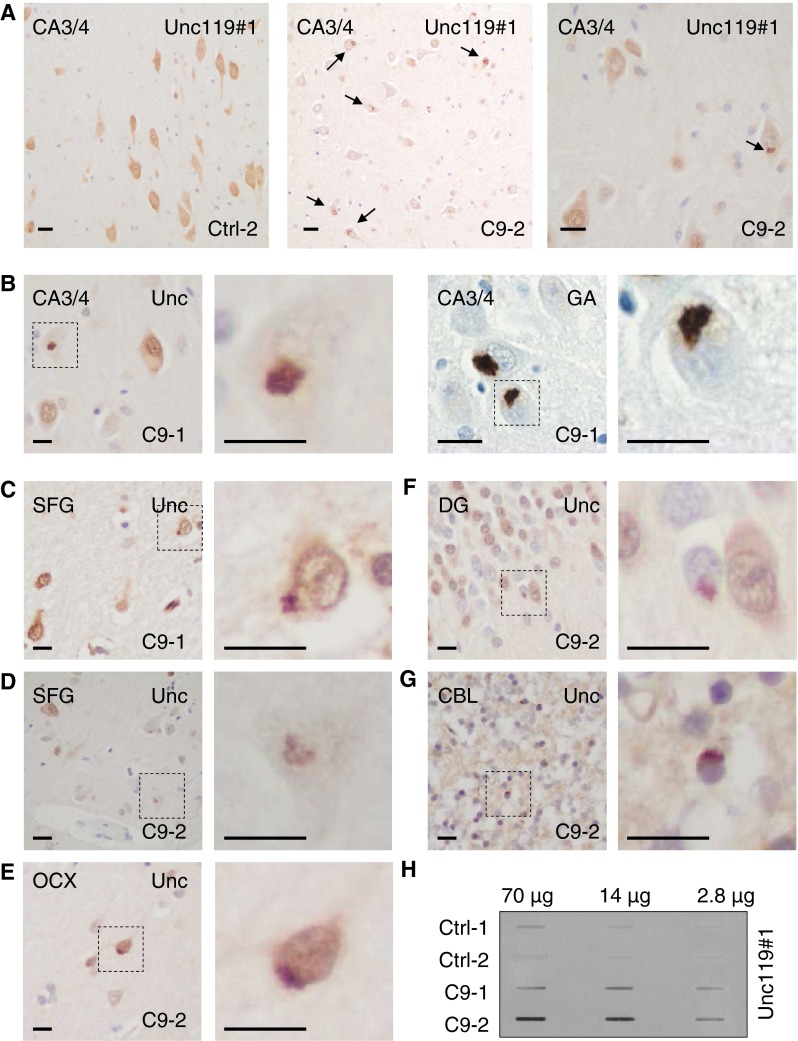
Unc119 forms neuronal cytoplasmic inclusions in *C9orf72* patients. **a**–**g** Immunohistochemistry for Unc119 in two *C9orf72* mutation carriers (C9-1 and C9-2) and a control case (Ctrl-2) using antibody Unc119#1. **a** Whereas Unc119 is distributed throughout the cytosol in hippocampal cornu ammonis regions 3/4 (CA3/4) of a control case, a *C9orf72* patient shows neuronal cytoplasmic inclusions. Scale bar represents 20 µm in overviews and 10 µm in close-up. **b** Unc119-positive inclusions have a similar shape as poly-GA inclusions. **c**, **d** In the superior frontal gyrus (SFG) Unc119-positive cytoplasmic inclusions are detectable in large neurons of mutations carriers. **e**–**g**) Further Unc119-positive neuronal cytoplasmic inclusions are found in the occipital cortex (OCX) and in the granular cell layers of the dentate gyrus (DG) and the cerebellum (CBL). In various areas, a fraction of cells with Unc119 inclusions shows a clear reduction of cytosolic Unc119 suggesting a redistribution of cytosolic Unc119 into aggregates (close-ups in **b**, **d**, **f**). *Scale bars* represent 10 μm. Counterstains in A-G were done with hemalum. **h** Filter trap assay detects insoluble Unc119 in 1 % SDS in the frontal cortex of mutation carriers, but not in healthy controls

Next, we analyzed Unc119 localization in *C9orf72* patients by immunohistochemistry using antibody Unc119#1 to validate co-aggregation with poly-GA found in vitro. In CA3/4 of the hippocampus Unc119 was mainly localized in the cytoplasm (Fig. 6a). Moreover, in all analyzed *C9orf72* FTLD/ALS patients Unc119-positive NCIs were detected, but no Unc119 NCIs were seen in healthy controls (Fig. 6, S8d). In the hippocampus of *C9orf72* patients, Unc119 formed star-shaped NCIs that appeared similar to poly-GA inclusions (Fig. 6b).

Further Unc119 NCIs were detectable in frontal cortex (Fig. 6c, d), occipital cortex (Fig. 6e) and the hippocampal dentate gyrus (Fig. 6f). Importantly, in a fraction of neurons nearly all Unc119 was sequestered into aggregates (Fig. 6b, d, f). Despite abundant DPR pathology only one of the five *C9orf72* cases showed prominent Unc119 NCIs in the cerebellum (Fig. 6g). The second Unc119 antibody (Unc119#2) appeared less sensitive but showed robust NCI pathology in the frontal cortex and in the dentate gyrus (Fig. S8a/b). With both antibodies no Unc119 inclusions were detected in control cases (Fig. 6a, S8c/d).

To further validate antibody specificity we performed competition experiments with GST-Unc119 using immunoblotting (Fig. S9a) and immunohistochemistry (Fig. S9b). Both soluble and inclusion staining were strongly reduced upon preincubation with purified GST-Unc119 further confirming specificity of the Unc119#1 antibody (Fig. S9b). Importantly, this antibody also detected insoluble Unc119 in *C9orf72* patients but not in controls using filter trap (Fig. 6h).

**Fig. 7:**
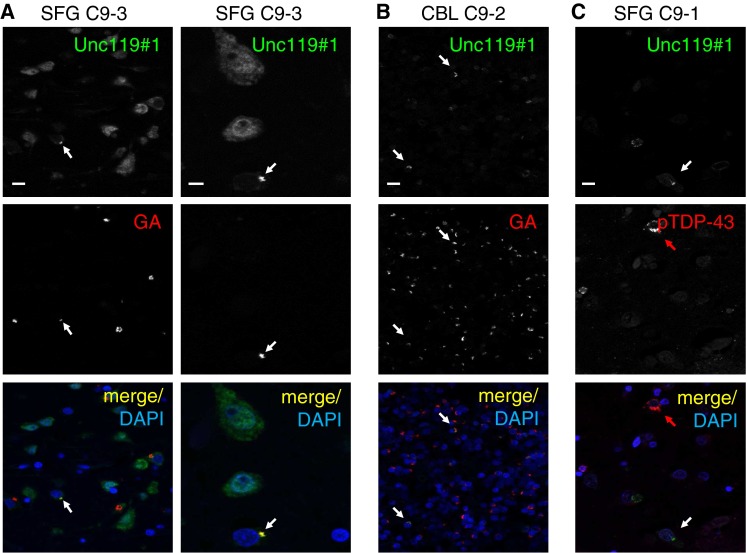
Unc119 co-aggregates with poly-GA, but not with TDP-43 in patients with *C9orf72* mutation. Double immunofluorescence analysis of Unc119 with poly-GA or phosphorylated TDP-43 (pTDP-43) in *C9orf72* mutation cases C9-1, C9-2 and C9-3. **a** In the superior frontal gyrus (SFG), a subset of poly-GA-positive neuronal cytoplasmic inclusions also contains Unc119. Redistribution of Unc119 compared to GA-negative cells can be seen in a fraction of co-aggregating cells (*white arrows*). **b** In the cerebellar granular cell layer (CBL) abundant cytoplasmic poly-GA inclusions are only rarely positive for Unc119 (*white arrows*). **c** As shown for the superior frontal gyrus, Unc119 (*white arrow*) and pTDP (*red arrow*) are not co-localized in the same cytoplasmic inclusions. *Scale bars* represent 10 μm for overviews and 5 µm for the close-up in the second column

Double immunofluorescence staining with both Unc119 antibodies confirmed co-localization of poly-GA and Unc119 in the cortex and cerebellum of *C9orf72* cases (Fig. 7a, b, S10a). Quantitative analysis in the frontal cortex of three FTLD/ALS patients revealed that Unc119 was present in 9.5 ± 2.7 % of GA inclusions (mean ± standard deviation >300 poly-GA inclusions counted per patient). In contrast, only 0.4–3.3 % of GA inclusions were Unc119 positive in the cerebellum (1.6 ± 1.5 %). In the occipital cortex an intermediate level of co-aggregation was observed (5.8 ± 1.6 %). All Unc119 inclusions were also poly-GA positive suggesting that DPRs drive inclusion formation. Importantly, despite abundant DPR and phospho-TDP-43 pathology in the frontal cortex, there was no co-localization of Unc119 and phospho-TDP-43 within inclusions (Fig. 6c, S10b).

Taken together, Unc119 specifically co-aggregates in poly-GA inclusions in *C9orf72* cases. Notably, Unc119 inclusions were preferentially detected in the frontal cortex, the main region for neurodegeneration in FTLD. Thus, region-specific Unc119 aggregation may contribute to the selective vulnerability of specific neuron populations to *C9orf72* repeat expansion in vivo.

## Discussion

Our work establishes a cell culture model for *C9orf72* FTLD/ALS that reproduces core findings in patients and directly links *C9orf72* repeat translation to neurodegeneration. Using quantitative analysis of the poly-GA interactome, we identified a novel co-aggregating protein, Unc119, which has been linked to axon maintenance in *C. elegans* previously [23, 30].

### DPR aggregation

Expressing DPR proteins from nearly GGGGCC-free synthetic genes containing ATG start codons allowed us to compare the aggregation properties of the five different DPR species while largely excluding potential secondary effects through RNA toxicity. Previous work with GGGGCC-based expression constructs did not lead to inclusion formation even when a start codon was present [55]. The higher expression levels in our system presumably accelerate disease mechanisms that would normally require gradual build-up of DPR proteins in the brain. In cell culture, the five DPR species displayed remarkably different properties. Only poly-GA expression resulted in compact cytoplasmic inclusions similar to those seen in *C9orf72* mutation brains [33, 36, 55] suggesting that it may be the main driving force for aggregation (Figs. 1, 2). This is in line with the observation that virtually all TDP-43-negative inclusions in *C9orf72* patients contain poly-GA, while antibodies against the other DPR species label only a fraction (10–50 %) of these inclusions [35, 36]. Interestingly, poly-GR and poly-PR formed mainly nuclear inclusions similar to the occasional nuclear DPR inclusions previously identified in patients with poly-GA and p62 antibodies [1, 29, 36]. These two charged DPR species might be actively imported into the nucleus, because a high density of positively charged arginines is also common in classical nuclear localization signals [14]. The discrepancy between aggregation properties observed in patients and our cell culture might be due to the fact, that the synthetic DPR proteins used are much shorter than the several hundred or even thousand repeats found in patients [5, 51].

### DPR toxicity

How *C9orf72* repeat expansion leads to neurodegeneration is poorly understood. In fly models RNA toxicity from a 30-mer repeat seems to be the main cause of neurodegeneration [52]. Neurons derived from *C9orf72* patients show normal viability, but increased sensitivity to cellular stressors [2, 15, 43]. Zu and colleagues reported combined RNA and protein toxicity for poly-PR and poly-GP in non-neuronal cell culture in the absence of inclusion formation [55]. Despite robust DPR expression in transfected HEK293 cells, we found no evidence for cell death due to protein toxicity in an LDH release assay with the five DPR species. Moreover, the GGGGCC expression constructs without ATG start codon and GGGGCC repeat based poly-GP construct were not toxic, suggesting HEK293 cells are not overtly sensitive to either *C9orf72* repeat RNA or protein toxicity under our conditions. We could not analyze GGGGCC repeat toxicity in neurons, because the repeat seems to block lentiviral packaging. In contrast, caspase activation and DNA fragmentation suggest that p62-positive poly-GA inclusions lead to apoptosis in primary hippocampal and cortical neurons (Figs. 2, 3). Since the synthetic poly-GA gene largely lacks GGGGCC repeats and requires an ATG start codon to cause toxicity, DPR proteins themselves can cause toxicity in neurons. Due to our construct design these findings, however, do not rule out additional or synergistic effects through GGGGCC repeat-mediated RNA toxicity or *C9orf72* haploinsufficiency in the pathogenesis of *C9orf72* FTLD/ALS. Due to the resemblance of poly-GA aggregates in neurons and patients we focused our study on poly-GA toxicity in neurons. However, it would also be interesting to analyze the effects of other DPR species alone or in combination with poly-GA in neuron culture.

Overexpression models have been invaluable tools to study neurodegenerative diseases but abnormally high levels of the aggregating proteins could also complicate the interpretation [16]. Importantly, lentiviral transduction in our system led to poly-GA aggregates that were comparable in size and poly-GA levels to inclusions from patients suggesting that the observed toxicity of poly-GA in cultured cells is also relevant in vivo (Fig. S3).

### Poly-GA interactome

Revealing the interaction profile of poly-GA is an important step to understand the mechanisms leading to the DPR toxicity. Novel instruments, advances of proteomics workflows and new bioinformatics algorithms have greatly increased the accuracy and depth of analysis as well as number of applications for quantitative proteomics [3, 6, 37]. Using GA_149_-GFP expression, we identified several interacting proteins by affinity purification and quantitative proteomic analysis (Table 1). However, we cannot exclude that additional proteins co-aggregate with the DPR inclusions in *C9orf72* patients. Importantly, the top hit was p62/SQSTM1, an ubiquitin-binding protein that is found in almost all types of intracellular protein aggregates in neurodegenerative diseases including DPR inclusions [1, 25, 36]. This validates our cell culture model and the unique potential of quantitative mass spectrometry to identify disease-relevant protein interactions. Additionally, we found several proteins associated with the ubiquitin proteasome system, but could not detect altered proteasomal expression or activity in poly-GA expressing HEK293 cells or neurons. Interestingly, a Gly/Ala-rich repetitive stretch of about 240 amino acids in EBNA1 was found to block its own proteasomal degradation suggesting that poly-GA may also interfere with the proteasome system [27]. However, in contrast to our findings with poly-GA, the Gly/Ala-rich region in EBNA1 prevents interaction with the proteasome [46], which may be explained by the distinct sequences. EBNA1 only contains GA monomers and dimers and does not form cellular inclusions.

Proteasomal dysfunction has been controversially discussed as a pathomechanism in poly-Q repeat disorders [45]. The poly-G aggregates derived from the CGG repeat expansion in FXTAS are also ubiquitinated [48]. Thus, the ubiquitin proteasome system is clearly linked to repeat expansion diseases although the mechanistic contribution to neurodegeneration remains unclear.

Apart from the ubiquitin–proteasome system, we detected co-localization of poly-GA inclusions with overexpressed and endogenous Unc119, which was among the identified poly-GA interacting proteins (Figs. 4, 5, 7, S10). In the brain, many neurons with Unc119 inclusions show little residual cytosolic Unc119 staining indicating that poly-GA inclusions in *C9orf72* patients lead to partial Unc119 sequestration (Figs. 6, 7). In cultured neurons, poly-GA expression strongly decreases the levels of soluble Unc119 suggesting a possible loss of function component in the disease in brain regions where it aggregates. Interestingly, we only scarcely detect Unc119 inclusions in the cerebellum, an area which shows little neurodegeneration in *C9orf72* patients despite abundant DPR pathology [29]. Unc119 has mainly been studied in the *C. elegans* nervous system and the mammalian retina. Importantly, Unc119 knockout in *C. elegans* almost completely paralyzes the worms and disturbs axonal development and maintenance [23, 30, 31]. Unc119 serves as a trafficking factor for myristoylated proteins, which it specifically binds through a hydrophobic pocket composed of β-sheets [8]. It is intriguing that the binding motif to Transducin α in the retina was mapped to the myristoylated N-terminal GAGASA sequence which strongly supports our interaction data with poly-GA [53]. Apart from this photoreceptor protein the only other known cargos in the nervous system are G_α_ proteins in the *C. elegans* olfactory system [8]. It will be important to elucidate how Unc119 sequestration affects neuronal function in *C9orf72* patients. We suspect that poly-GA enters and clogs the hydrophobic cavity of Unc119 and thus inhibits transport of so far unidentified myristoylated Unc119 cargos in cortical neurons, which may contribute to neurotoxicity observed upon Unc119 knockdown or poly-GA expression. An Unc119 nonsense mutation was found in a patient with cone rod dystrophy and causes retinal degeneration in mice [24], which is consistent with the toxicity we observed upon Unc119 knockdown in cortical and hippocampal neurons. Importantly, Unc119 overexpression partially rescues poly-GA toxicity in primary neurons, while Unc119 knockdown does not further increase poly-GA toxicity. Together this indicates that Unc119 sequestration is a major cause of poly-GA toxicity.

In conclusion, our data strongly suggest that the unusual translation of the expanded repeats into poly-GA causes neurodegeneration. Co-sequestration of crucial neuronal proteins, such as Unc119, within DPR aggregates may be a novel pathomechanism in *C9orf72* FTLD/ALS further strengthening the importance of DPR aggregates in disease context.

## Electronic supplementary material

Below is the link to the electronic supplementary material.

Supplementary material 1 (PDF 17185 kb)
