# Supplementary material for: C9orf72 FTLD/ALS-associated Gly-Ala dipeptide repeat proteins cause neuronal toxicity and Unc119 sequestration
Source: Acta Neuropathol. 2014 Aug 14;128(4):485–503. doi: 10.1007/s00401-014-1329-4 (PMC4159571; doi:10.1007/s00401-014-1329-4)
Supplement: Supplementary file 1 — Supplementary material 1 (PDF 17185 kb) [file 401_2014_1329_MOESM1_ESM.pdf]

Online Supplemental Materials

C9orf72 FTLD/ALS-associated Gly-Ala dipeptide repeat proteins cause neuronal toxicity and Unc119 sequestration

Stephanie May*, Daniel Hornburg*, Martin H. Schludi*, Thomas Arzberger, Kristin Rentzsch, Benjamin M. Schwenk, Friedrich A. Grässer, Kohji Mori, Elisabeth Kremmer, Julia Banzhaf-Strathmann, Matthias Mann, Felix Meissner, Dieter Edbauer#

Includes:

Supplemental Figures S1-S10

Supplemental Table S1

Supplemental Methods (with sequence information)

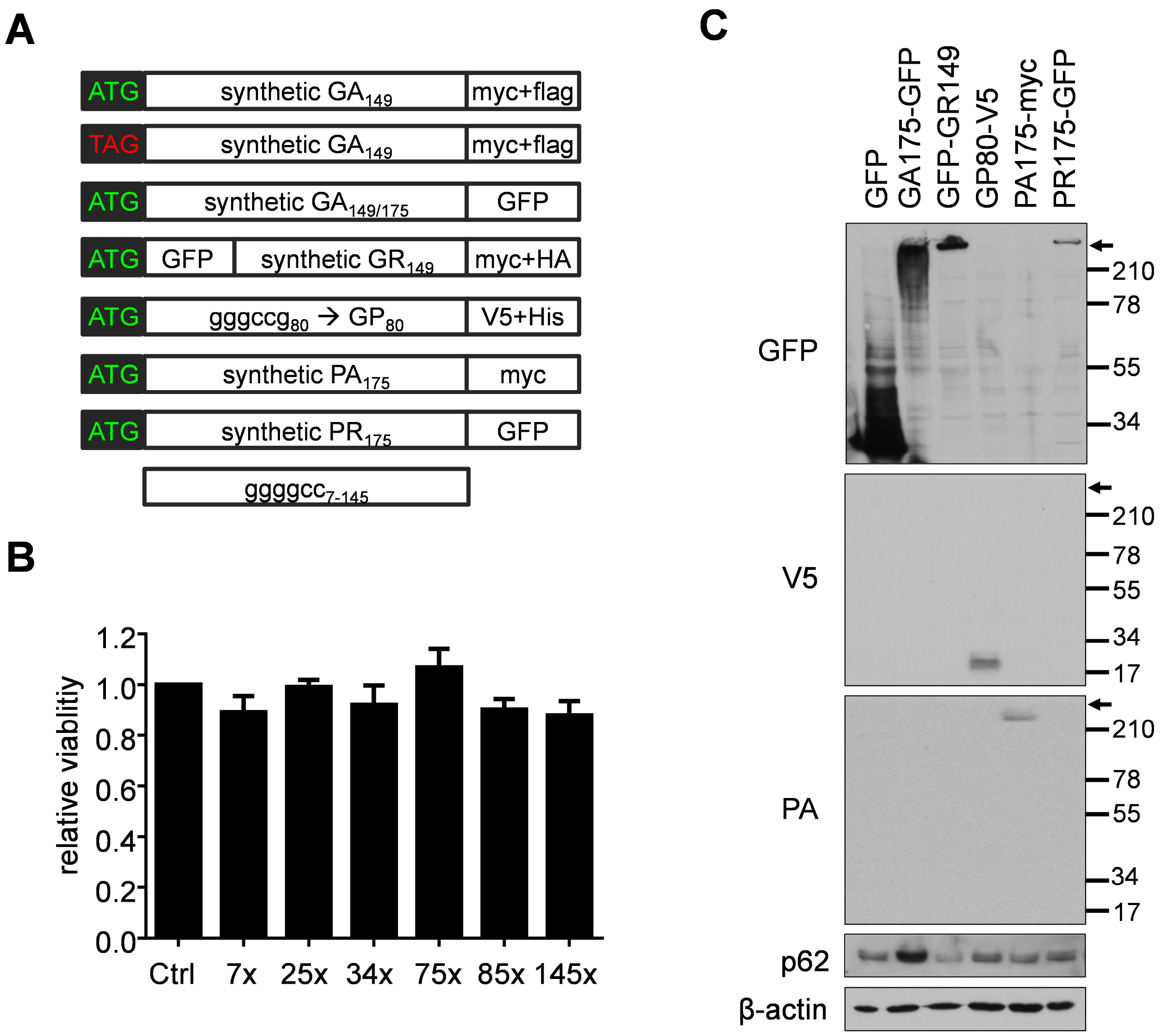

Fig. S1: Expression system for specific DPR proteins. (A) Illustration of the DPR-expressing constructs for poly-GA, -GR, -GP, -PA and -PR used in this study. For better expression and detection all constructs contain an ATG start codon and epitope tags (GFP, myc, flag, V5 or His). Poly-GA, -GR, -PA and -PR are expressed from nearly GGGGCC-free synthetic genes. Poly-GP is expressed from the endogenous repeat sequence. The different construct design was necessary to overcome expression difficulties with some constructs. For example, GR_{149}-GFP showed no detectable expression, while GFP-GR_{149} is expressed nicely. PA_{175}-GFP transfection resulted in expression of PA_{175}-GFP and free GFP (presumably through internal initiation), which precludes analysis by immunofluorescence. Unfortunately, several attempts for gene synthesis of poly-GP failed and we had to resort to GGGGCC-based expression. For control experiments a TAG-GA_{149}-myc construct and several GGGGCC repeat constructs lacking ATG start codons were used. (B) Cell viability of HEK293 cells transfected with

GGGGCC-repeat expressing constructs of increasing length (lacking an ATG start codon) was measured by XTT assay on day 3. No significant toxicity compared to an empty vector control (Ctrl) was observed (one-way ANOVA, Dunett's post-test). (C) HEK293 cells were transfected with the five different poly-DPR constructs (GA_{175}-GFP, GFP-GR_{149}, GP_{80}-V5, PA_{175}-myc and PR_{175}-GFP) or GFP as in Figure 1. Immunoblotting with indicated antibodies shows expression of DPR species and control GFP in transfected HEK293 cells. GA_{175}-GFP, GFP-GR_{149}, PA_{175}-myc and PR_{175}-GFP are detected at the top of the gel (arrow). p62 is upregulated in poly-GA expressing cells. β-actin was used as a loading control.

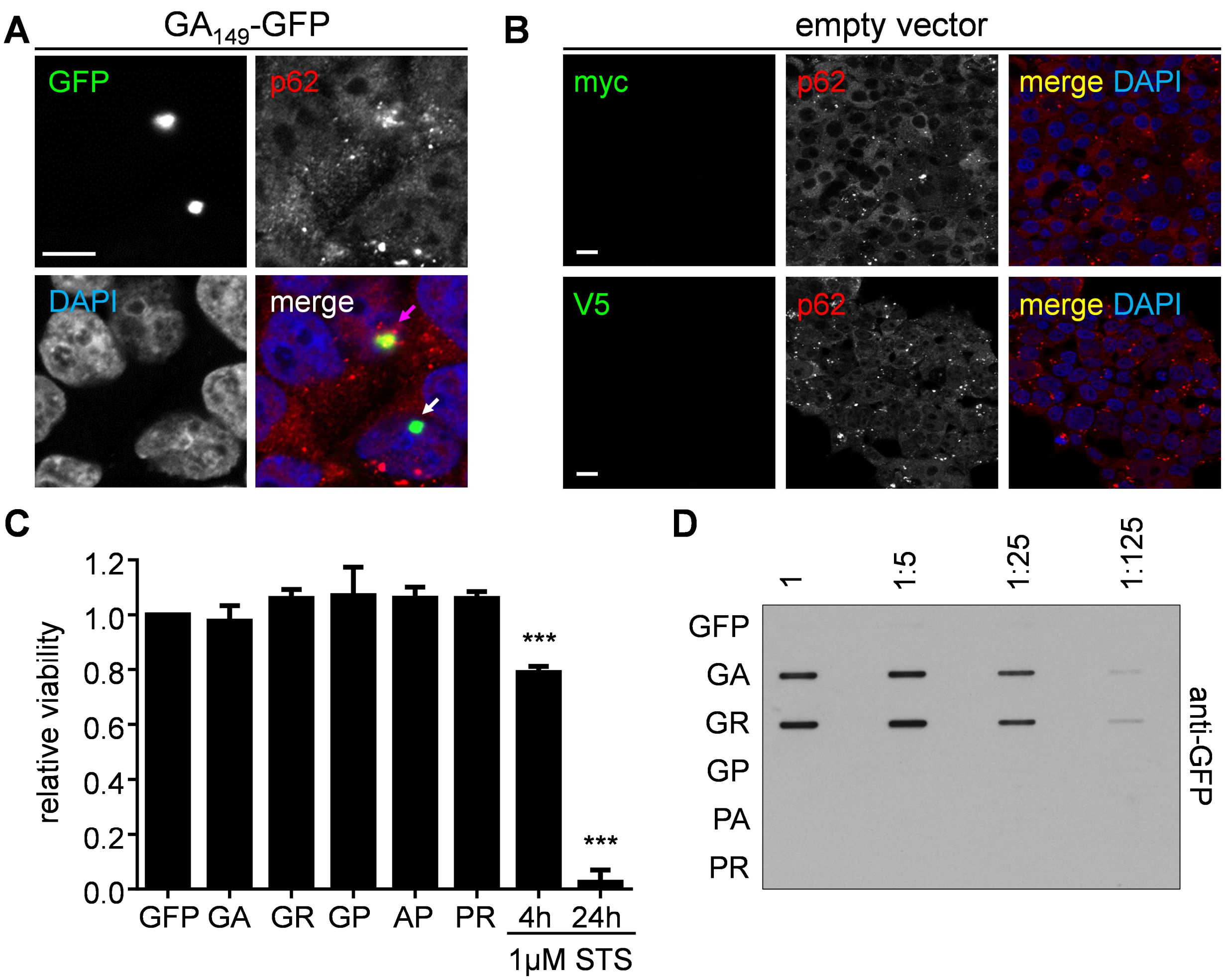

Fig. S2: DPR proteins differentially aggregate in HEK293 cells without inducing cell death. HEK293 cells were transfected with GFP, GA_{175}-GFP, GFP-GR_{149}, GP_{80}-V5, PA_{175}-myc and PR_{175}-GFP or empty vector as control for 3 days. (A) A fraction of nuclear GA_{175}-GFP inclusions (magenta arrows) is p62-positive. Scale bar 5 µm. (B) Immunofluorescence analysis as in Figure 1 shows that myc and V5 antibodies are specific. Scale bar 15 µm. (C) LDH release assay detects no significant toxic effect upon DPR expression compared to GFP control in HEK293 cells. Treatment with 1 µM Staurosporine was used as a positive control. n=3 experiments with 10 replicates each; mean ± SD, *** $p<0.001$ in one-way ANOVA with Dunett's post-test. (D) Filter trap assay for detection of insoluble protein in HEK293 homogenates containing 2% SDS. Immunodetection with anti-GFP. Aggregation of PA_{175}-myc and GP_{80}-V5 was not detectable with anti-PA and anti-V5 (not shown).

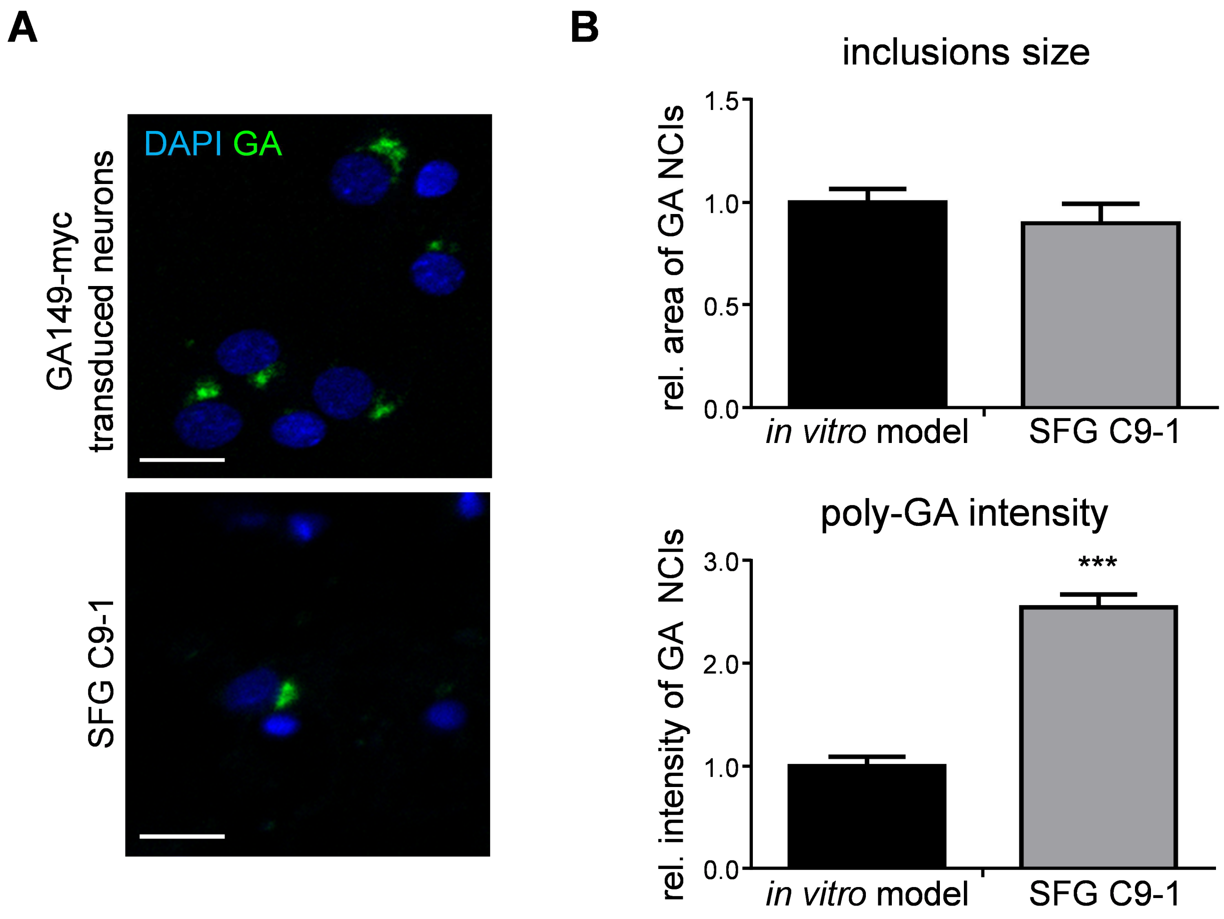

Fig. S3: Lentiviral poly-GA expression levels in primary neurons are comparable to *C9orf72* patients.

Immunostaining of primary cortical neurons transduced with GA_{149}-myc (DIV6+17) and a thin smear of cortical tissue (superior frontal gyrus from patient C9-1) under identical conditions with poly-GA antibodies. In the *in vitro* system a larger fraction of cells shows poly-GA inclusions than in patient brain (A) Scale bar depicts 20 µm. The poly-GA aggregates have similar size in both settings, but the integrated intensity of poly-GA staining is about two-fold higher in the inclusions in patients. Poly-GA aggregate area and intensity was manually quantified for 50 inclusions of each condition with ImageJ. Mean ± SD, Student's t-test, *** $p<0.001$(B). Thus, lentiviral transduction might even underestimate poly-GA toxicity.

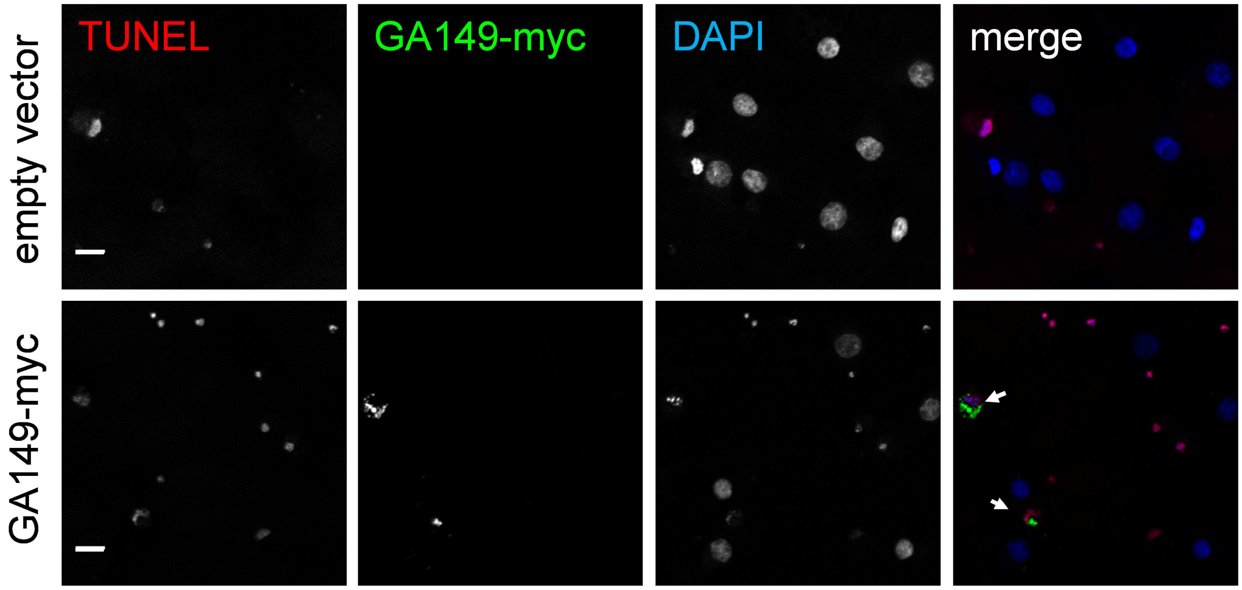

Fig. S4: TUNEL assays detects neuronal apoptosis upon poly-GA expression.
Primary hippocampal neurons transduced with GA_{149}-myc or empty vector (DIV6+17). TUNEL assay reveals apoptotic DNA fragmentation. Co-staining for poly-GA and nuclei. GA_{149}-myc transduces neurons show ~2.5 fold increased apoptosis (arrow). Quantification is shown in Figure 3C.

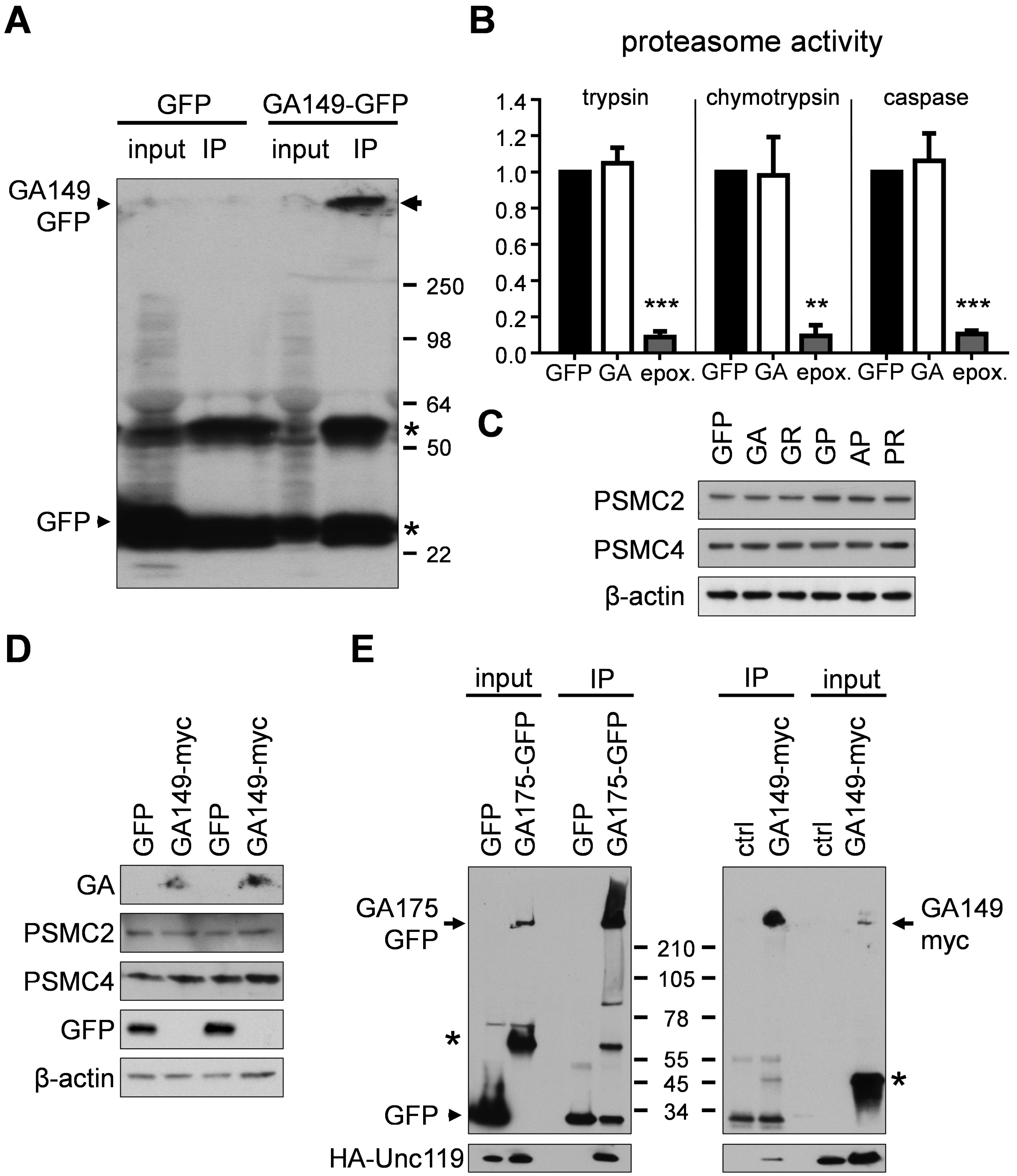

Fig. S5: Immunoprecipitation of poly-GA aggregates for LC-MS/MS analysis

(A) Primary cortical neurons (DIV6+17) transduced with either GFP or GA_{149}-GFP lentivirus and subjected to anti-GFP immunoprecipitation. Samples were used for quantitative mass spectrometry analysis (see Figure 4A and Table 1). Immunoblotting with anti-GFP confirms immunoprecipitation of GFP and GA_{149}-GFP (arrowheads). Asterisks indicated immunoglobulin heavy and light chain. Arrow indicates top of gel. (B) Expression of GA_{175}-GFP did not affect the

chymotrypsin-like, trypsin-like and caspase-like protease activity of the proteasome in HEK293 cells compared to GFP control (n=3 experiments with 6 replicates each, mean ± SD, no significant change in one-way ANOVA). The proteasome inhibitor epoxomycine (8 µM, 2 hours) significantly blocks proteasome activity in GFP-transfected cells (one-way ANOVA with Tukey's post-test, *** $p<0.001$; ** $p<0.01$). (C) Expression of proteasomal subunits (PSMC2, PSMC4) was not affected by expression of DPRs in HEK293 cells (GA_{175}-GFP, GFP-GR_{149}, GP_{80}-V5, PA_{175}-myc and PR_{175}-GFP). (D) Expression of proteasomal subunits (PSMC2; PSMC4) was unchanged in GA_{149}-myc transduced cortical neurons compared to GFP transduced controls. Three separate transductions are shown (DIV6+17). (E) Coimmunoprecipitation of Unc119 and poly-GA. HEK293 cells cotransfected with HA-Unc119 and GA_{175}-GFP or GA_{149}-myc for 3 days. The poly-GA proteins were immunoprecipitated with GFP or myc antibodies. Note that in freshly prepared protein extracts monomeric poly-GA can be resolved when directly loaded (asterisks). Aggregated poly-GA is stuck at the top of the gel (arrow).

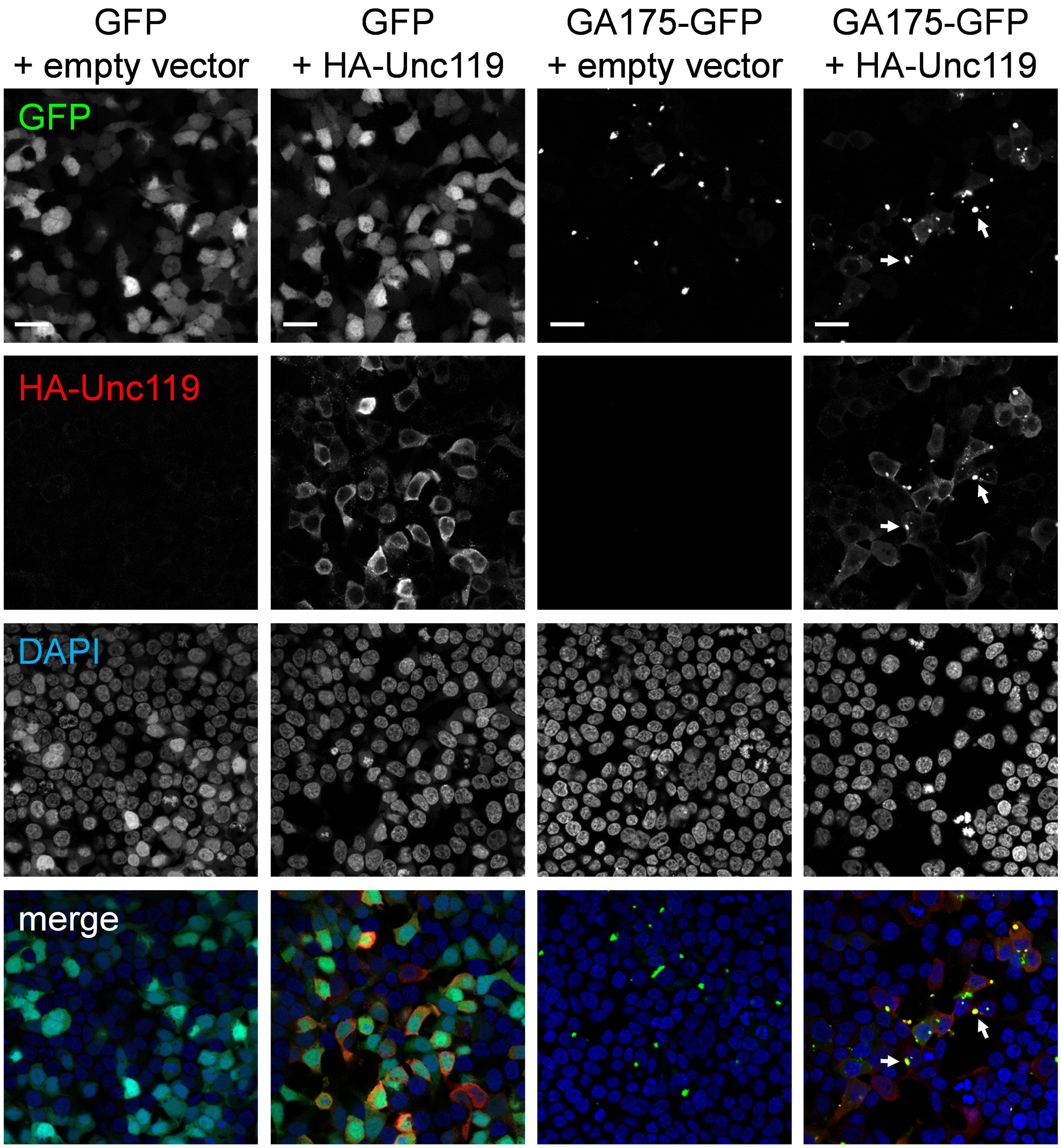

Fig. S6: Unc119 co-aggregates with poly-GA in HEK293 cells.

Immunofluorescence of HEK293 cells co-transfected with GFP or GA_{175}-GFP and HA-Unc119 or empty vector. GFP fluorescence, anti-HA immunostaining and DAPI as nuclear marker. Compare the HA-Unc119 localization in column 2 and 4. Many GA_{175}-GFP inclusions show co-aggregation of HA-Unc119 (examples marked with arrows). This figure shows separate channels of the images shown in Figure 4B.

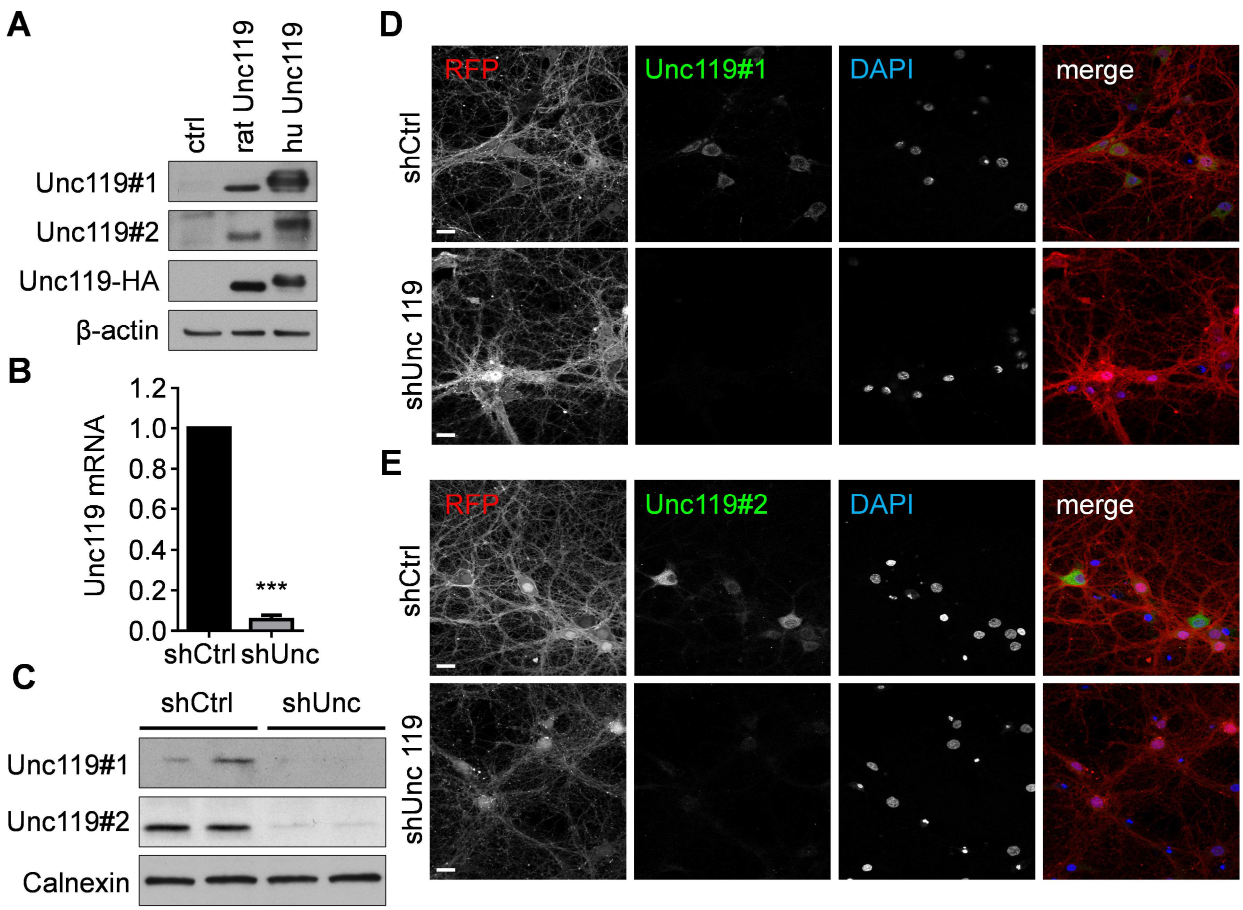

Fig. S7: Unc119 antibodies #1 and #2 are specific.

(A) Unc119 antibodies #1 and #2 detect rat and human HA-Unc119 overexpressed in HEK293 cells. Note that HEK293 cells show very little endogenous Unc119. (B-E) Primary cortical neurons were transduced with a shRNA targeting Unc119 or a control shRNA (DIV7+10). (B) RT-qPCR shows efficient reduction of Unc119 mRNA normalized to the reference gene GAPDH. mean ± SEM. N=3. $p<0.001$ in Student's t-test. (C) Two Unc119 antibodies (#1 and #2) show reduced Unc119 protein levels upon Unc119 shRNA transduction compared to controls in immunoblots. Two separate transductions are loaded. (D, E) Both antibodies detect reduced Unc119 protein levels by immunostaining. tagRFP co-expressed from the shRNA lentivirus shows high transduction efficiency. Scale bars represent 20 µm.

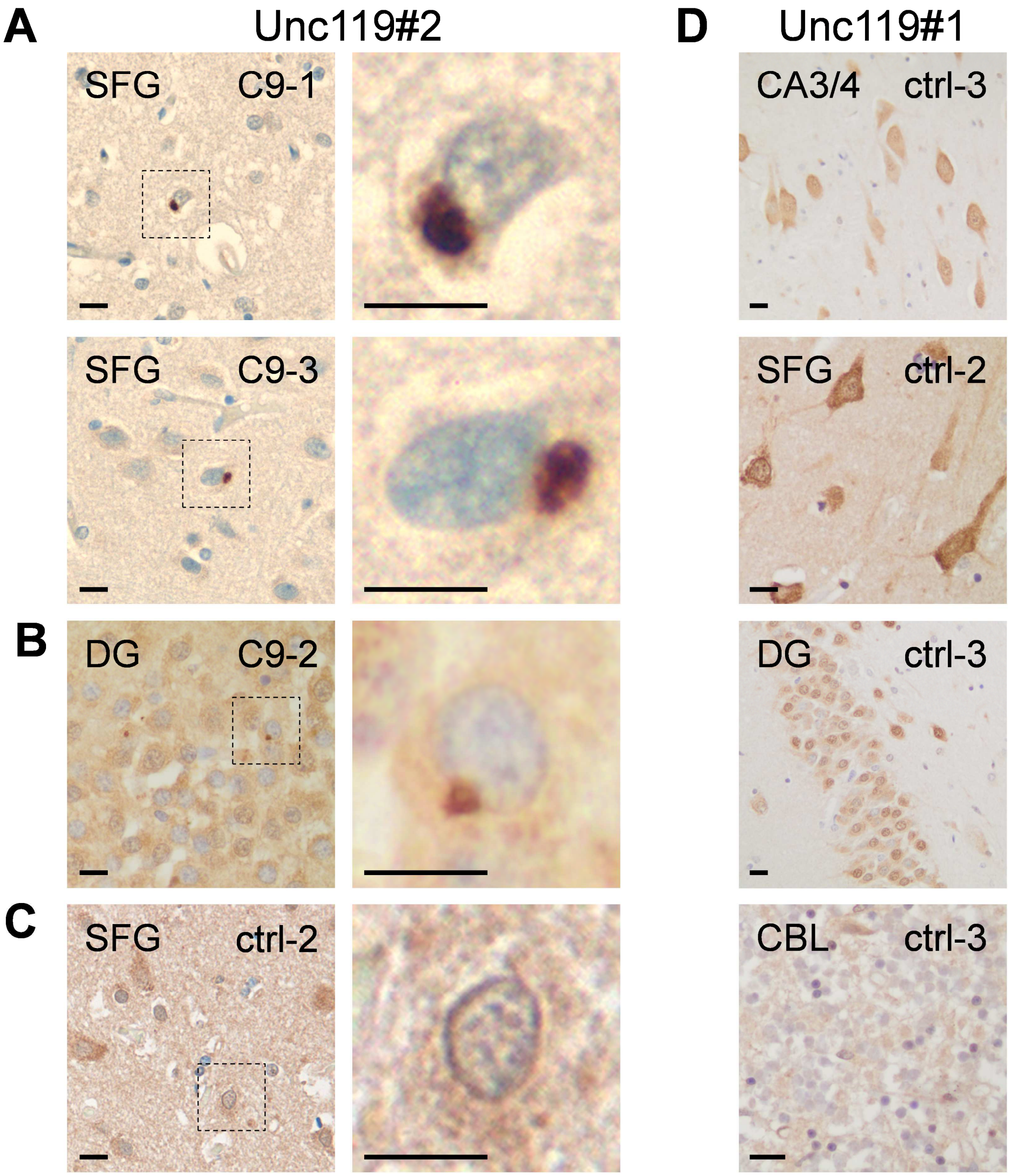

Fig. S8: Unc119 neuronal cytoplasmic inclusions in *C9orf72* patients.

(A-C) Immunohistochemistry for Unc119 in three *C9orf72* mutation carriers (C9-1, C9-2 and C9-3) and a control case (Ctrl-2) using Unc119#2 antibody. In mutations carriers Unc119-positive cytoplasmic inclusions are detectable in large neurons in the superior frontal gyrus (SFG) (A) and the dentate gyrus region of the hippocampus (B). No Unc119 inclusions are found in a healthy control (C). (D) Using antibody Unc119#1 no inclusions are seen in hippocampal cornu ammonis regions 3/4 (CA3/4), in the superior frontal gyrus (SFG), the dentate gyrus (DG) as well as in the cerebellar (CBL) granular cell layer of control cases (Ctrl-2 and 3). Counterstains were done with haemalum. Scale bars represent 10 µm. Stainings shown in (A) and (B) confirm the findings seen for Unc119#1 antibody (Figure 6).

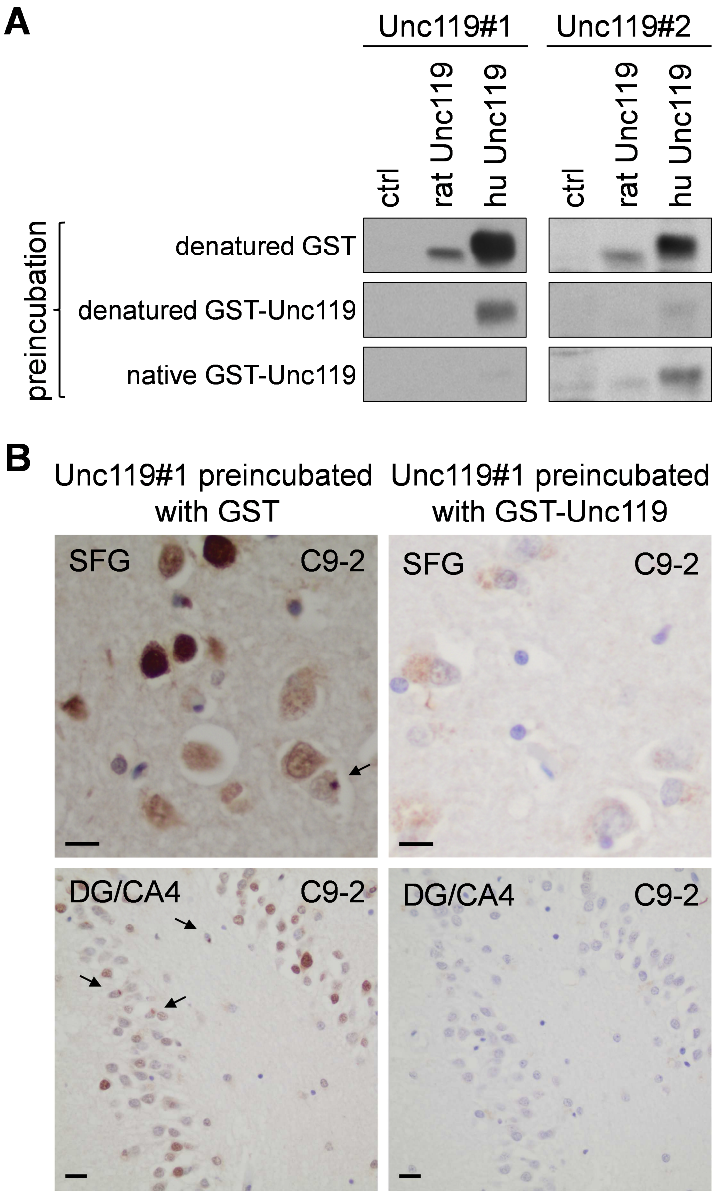

Fig. S9: Antigen preincubation confirms specificity of Unc119 staining in patient material. (A) Immunoblots of HEK293 cells transfected with rat and human HA-Unc119 using antibodies Unc119#1 and #2. To confirm specificity Unc119 antibodies were preincubated with 25 µg/ml native or denatured GST-Unc119 or denatured GST as a control. While specific Unc119#2 signal is best blocked with denatured GST-Unc119, the Unc119#1 antibody is best blocked with native GST-Unc119, which may explain the better sensitivity of Unc119#1 for immunohistochemistry. (B) Using the Unc119#1 antibody preincubated with GST-Unc119 but not with GST alone abolishes the staining of cells and inclusions strongly indicating antibody specificity for immunohistochemistry as shown for the superior frontal gyrus (SFG) and the hippocampal dentate gyrus/cornu ammonis region 4 (DG/CA4) of a *C9orf72* mutation carrier. Scale bars depict 10 µm for SFG and 30 µm for DG/CA4.

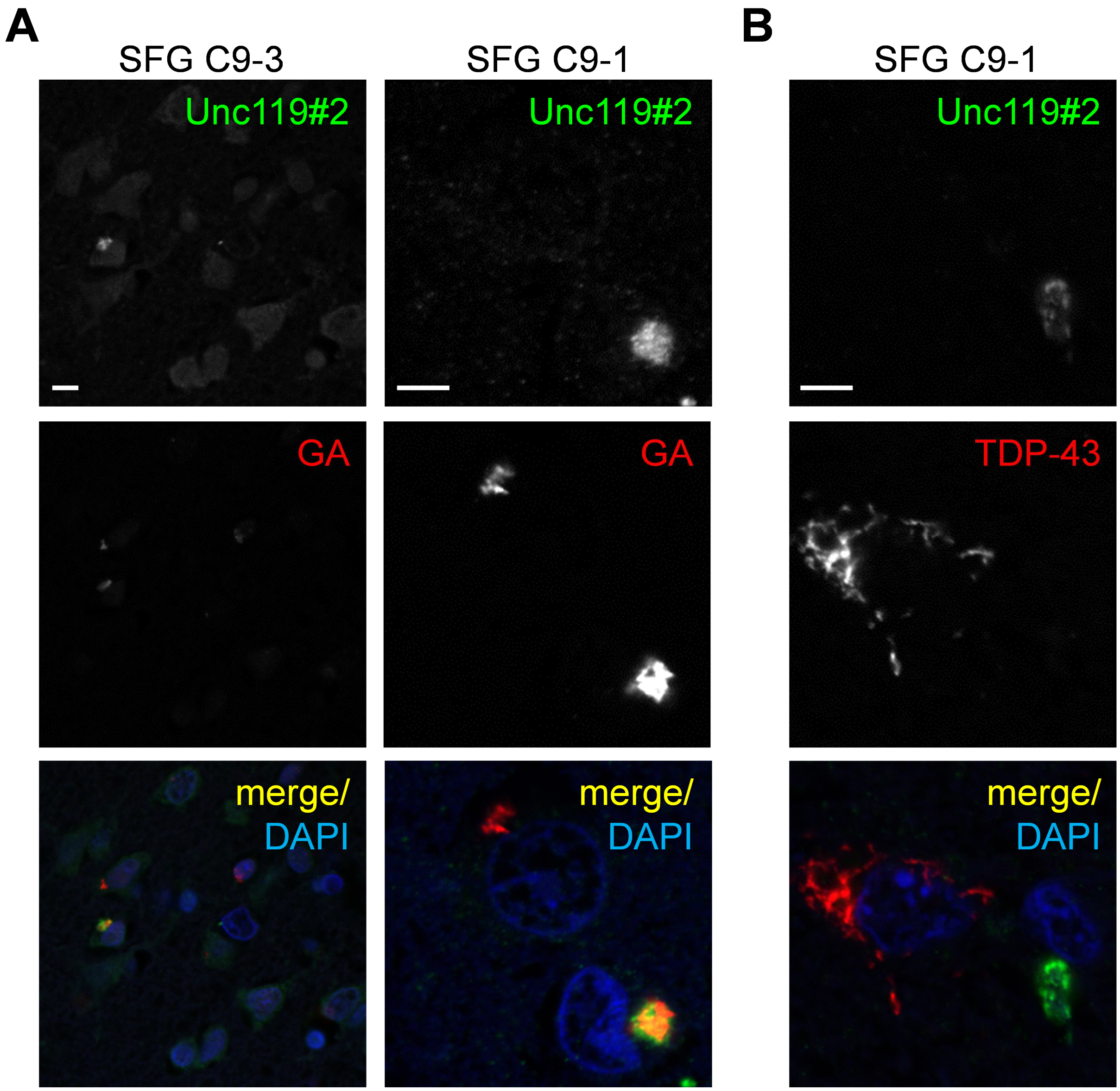

Fig. S10: Unc119 co-aggregation with poly-GA detected with second Unc119 antibody. Double immunofluorescence analysis of Unc119 with poly-GA or phospho-TDP-43 in *C9orf72* mutation cases C9-1 and C9-3. (A) In the superior frontal gyrus (SFG), a subset of poly-GA positive NCI also contains Unc119. (B) There is no co-localization of poly-GA and TDP-43 in the frontal cortex. Scale bars represent 10 µm in overviews and 5 µm in close ups. These staining confirm the findings with Unc119#1 antibody (Figure 7).

Table S1 Clinical information of human brain samples

Case number	GGGGCC expansion	Gender	Clinical and neuropathological diagnosis	Age at death
**Ctrl-1**	-	female	control, no neurological or psychiatric disease	47
**Ctrl-2**	-	male	control, no neurological or psychiatric disease	60
**Ctrl-3**	-	female	control, no neurological or psychiatric disease	60
**C9-1**	+	female	FTD/ALS	65
**C9-2**	+	female	FTD/ALS	59
**C9-3**	+	female	ALS/beginning FTD	47
**C9-4**	+	male	FTD/Parkinson	65
**C9-5**	+	female	ALS	63

Supplemental Methods

Sequence of the open reading frames from the synthetic DPR expression constructs. The EGFP sequences is shown in green.

GA175-GFP

atgggagctggtgctggtgcaggcgctggggcaggggctggcgctggtgccggtgctggtgccggcgcagg
cgctggtgctggtgctggtgctggggctggtgcaggcgcaggggctggtgcaggggcaggcgcaggcgctg
gtgctggcgcaggggcaggcgctggggctggtgcaggcgctggtgctggcgctggtgcaggcgctggcgct
ggtgctggcgctggcgcaggggcaggcgcaggcgcaggggctggtgctggtgccggtgcaggggctggggc
tggtgctggtgctggcgcaggcgctggtgcaggcgctggggctggcgcaggggctggtgctggtgcaggcg
ctggtgctggtgccggtgccggtgctggtgcaggggctggtgctggggctggggctggtgctggtgcaggg
gcaggggctggtgctggtgcaggggctggtgccggtgctggtgcaggggcaggggcaggcgcaggcgctgg
ggcaggcgctggtgcaggggcaggcgctggggcaggcgcaggcgcaggcgctggtgcaggggctggtgctg
gtgcaggcgcaggcgcaggggctggggctggcgctggtgctggtgctggggctggcgctggtgccggtgcc
ggtgcaggcgctggtgccggtgcaggggctggtgcaggcgctggtgctggtgcaggggctggtgctggtgc
tggtgccggtgctggcgctggtgctggggcaggcgctggcgctggggcaggggcaggggctggcgctggtg
ctggtgcaggcgcaggcgctggtgccggtgctggcgcaggcgctggtgctggtgcaggggctggcgcaggc
gctggcgcaggggctggtgcaggcgctggtgccggggcaggcgcaggggctggtgctggtgctggtgctgg
cgctggtgcaggggctggtgccggtgcaggcgctggtgcaggggctggggctggtgctggcgctggggctg
gtgcaggggctggggcaggcgcaggggctggtgccggtgcaggcgcaggggcaggcgcacccatggtggct
agaatggtgagcaagggcgaggagctgttcaccggggtggtgcccatcctggtcgagctggacggcgacgt
aaacggccacaagttcagcgtgtccggcgagggcgagggcgatgccacctacggcaagctgaccctgaagt
tcatctgcaccaccggcaagctgcccgtgccctggcccaccctcgtgaccaccctgacctacggcgtgcag
tgcttcagccgctaccccgaccacatgaagcagcacgacttcttcaagtccgccatgcccgaaggctacgt
ccaggagcgcaccatcttcttcaaggacgacggcaactacaagacccgcgccgaggtgaagttcgagggcg
acaccctggtgaaccgcatcgagctgaagggcatcgacttcaaggaggacggcaacatcctggggcacaag
ctggagtacaactacaacagccacaacgtctatatcatggccgacaagcagaagaacggcatcaaggtgaa
cttcaagatccgccacaacatcgaggacggcagcgtgcagctcgccgaccactaccagcagaacacccccа
tcggcgacggccccgtgctgctgcccgacaaccactacctgagcacccagtccgccctgagcaaagacccc
aacgagaagcgcgatcacatggtcctgctggagttcgtgaccgccgccgggatcactctcggcatggacga
gctgtacaagtaa

GA149-myc

Atgggagctggcgcaggcgctggggcaggggctggcgccggtgccggcgcaggggcaggcgcaggggctgg
ggctggggcaggcgctggcgctggcgcaggggctggggcaggcgcaggggcaggcgctggcgcaggggccg
gcgccggggccggggcaggcgctggggcaggggcaggggctggcgcaggcgcaggcgctggggctggcgct
ggcgcaggcgcaggcgcaggcgctggggcaggggctggggctggggccggcgccggggccggcgctggggc
tggcgcaggcgctggcgccggggccggggccggcgcaggcgctggcgctggggcaggcgcaggggcagggg
caggcgctggcgctggcgctggcgcaggggctggcgctggggctggggcaggggcaggggctggggctggc
gctggggctggggctggcgctggcgcaggggcaggggctggggcaggggcaggcgcaggggccggcgccgg
ggccggcgctggcgcaggggctggcgcaggcgctggggctggcgcaggcgcaggggcaggcgctggggctg
gcgccggggccggggctggggccggcgccggggccggggcaggggcaggcgcaggcgcaggggcaggggca
ggcgcaggggctggcgcaggggcaggggccggcgccggggccggcgcaggggccggcgccggggccggggc
cggggctggggctggggctggcgcaggggcaggcgcaggggctggcgcaggcgctggcgcaggggcaggcg
caggcgcaggcgcaggggctggggctggcgcaggcgccggggccggggctggcgctggcgcaggcgctggg
gcaggcgcaggcgctggggctggggcaggggctggcgctggggcttggagtggcagagctagaggaagggc
tagaggcggcgctgctgtggctgtgcctgctccagctgctgctgaagctcaggctgtggccagcggctcta
gaagcgcttggagccacccccagttcgagaagctggaagaacagaagctgatctccgaagaggacctgggc
agcgactacaaggacgacgacgacaaatga

GFP-GR149

atggtgagcaagggcgaggagctgttcaccggggtggtgcccatcctggtcgagctggacggcgacgtaaa
cggccacaagttcagcgtgtccggcgagggcgagggcgatgccacctacggcaagctgaccctgaagttca
tctgcaccaccggcaagctgcccgtgccctggcccaccctcgtgaccaccctgacctacggcgtgcagtgc
ttcagccgctaccccgaccacatgaagcagcacgacttcttcaagtccgccatgcccgaaggctacgtcca
ggagcgcaccatcttcttcaaggacgacggcaactacaagacccgcgccgaggtgaagttcgagggcgaca
ccctggtgaaccgcatcgagctgaagggcatcgacttcaaggaggacggcaacatcctggggcacaagctg
gagtacaactacaacagccacaacgtctatatcatggccgacaagcagaagaacggcatcaaggtgaactt
caagatccgccacaacatcgaggacggcagcgtgcagctcgccgaccactaccagcagaacacccccatcg
gcgacggccccgtgctgctgcccgacaaccactacctgagcacccagtccgccctgagcaaagaccccaac
gagaagcgcgatcacatggtcctgctggagttcgtgaccgccgccgggatcactctcggcatggacgagct
gtacaagaagcttgccaccatgggacgcggccgcggaagaggcagaggaaggggaggggcagggggcgag
gcaggggacggggacgaggaagagggcgcggacgggggagaggacggggcaggggaagaggaaggggcaga
ggccgcggaaggggaagggggagaggaagaggacgaggaagaggccgaggcaggggcagagggcgagggcg
aggaaggggaagaggcagaggacgcggccgaggccgaggacgcggaagaggaagaggccggggaagaggca
ggggacgaggcagaggcaggggaaggggaagaggacggggacgggggcgaggcagaggaagaggccgagga
agaggacggggaaggggacgcggacgaggcagggggagaggcaggggcaggggcagaggcagaggacgagg
gcggggacgcggaaggggacgaggaaggggaaggggacgggggagggggaggggaagaggaagaggcaggg
gcagaggacgcggcagagggcggggaagaggcagaggcagaggaagaggacgaggccgcggaagggggcga
gggcggggacgaggacgcggacgcggcagaggaagaggcagaggccggggacgaggacgaggaaggggcag
aggacgaggacgaggcagaggaagaggacgcggcagggggagaggacgaggacgcggacgaggacggggaa
gaggaagggggagaggaaggggcagaggcaggggacgggggaggggccgaggacggggccgaggcaggggg
cgaggaagaggaagaggaaggggacggggacggggcagaggccgaggccgcggacggggcaggggacgcgg
aagaggacgcggcagaggcagaggaagaggaaggggaaggggcagaggaagggggagaggacgcggagtcg
tgggagctggacctggcgcaggacctggaagaggatgtggctgtggcgcctgtgctagaggcggcggaggc
gctgggggcggagaatgggtgtcagaggaagccgcctcttggagagtggccgtgtggggatctgccgctgg
caagagaagaggctctagaagcgcctggtcccacccccagttcgagaagctcgaggaacagaagctgatct
ccgaagaggacctgggcagctacccctacgacgtgccagactacgcctga

AP175-myc

atgcctgcccctgctccagctcctgcaccagcaccagctcccgctccagcgcctgctcctgcccccgcacc
cgccccagcccctgcaccggcgcctgctccagctcccgctcctgctcccgctccagcaccggcccctgcac
cagccccagcgccagctccggctccagctccagccccagctcctgcgccagctcctgctcctgctcctgcc
cctgcgcctgctccagcaccagcccctgcccctgctcctgcaccggctccagctccagctcctgcgcctgc
acccgcaccagcgccagcaccagcaccagctccagcccctgctccagcaccagcaccggcaccagctccgg
caccagcaccagcgcctgcaccagctcccgctccagctcctgcaccagctccagcaccagcgcctgctcca
gcaccagcgccagctccagctccagcacccgcaccagcaccagcaccagctcccgctcccgcaccagcccc
tgctccggctccggctcctgctcctgctccggctcctgcaccagcccctgctccagctccagctccagcgc
cagcaccagctccagctccggctccagctcctgctcctgcaccagcccccgcacccgcacccgcacccgct
ccagcgcccgctcctgcaccagctccagcgcctgctccagctcctgcccctgctccagcccctgcccccgc
tcctgcgcctgctcctgctccagctccagctcccgctcctgccccagccccagcaccagctccagcaccag
cccctgcacccgcccctgcaccagcacccgctccagcaccagctccggctccagcacccgctccagctcca
gcaccagcacccgcccctgctccagctccagcccctgctccggctcctgctcccgcaccggcacccgctcc
agctccagctcctgctcctgctcctgctccagcacccgctcccgcaccggcgccagcccctgctcctgccc
cagctccagctcctgcgccggctccagcgcctgcaccagcaccggcaccagcccctgctagcgaacagaag
ctgatcagcgaagaggacctctga

PR175-GFP

atgcccagacccagacctagaccaaggcctaggccaagacctcgccctcgaccaagacctcgtccaagacc
tagacctagacccagaccacgccctagacctcgaccacgtcctaggcctagaccaagaccaagaccccgac
caaggccacgtcctagaccacgtcccagaccaaggccaagacctagaccaagaccgaggcccaggcctaga
cctcgacctagacctagaccaagacctcggccccgtccacgaccaagaccaagacctagacctaggccacg
acccagacctcgtcctcgtcctagaccaagacctaggcctcgacctaggccaaggcctcgtccaagaccaa
gaccaaggccacgcccacgtcctagacctaggcctagacctcgtccacggccacgacctagacctcgacca
agaccaagacccaggccaagaccaagaccacggcctagaccacgacctaggccacgtccaagaccccgacc
tagaccaagacccagacctcgaccacgcccaagacctaggcctcgtcccagacctagacccagaccaagac
ccagaccccgtccaagacctaggccaaggccaaggcctagacccaggcctagaccacgtcctcgtcctaga
cctcggcctaggccaagacctagacctcgtcctagaccgcggcctcgacctagacccagacctagacctag
acctcgcccaaggccacgtccaaggcccagaccacgtcctagaccaagacctcgccctaggccaaggccaa
gaccacgaccaagacctcggcctagaccccgtccaagaccaagacctcgaccaagaccaaggcctcgccct
agaccacgaccacgaccccgtcctaggcccagacctaggccaagaccacgacctaggcctagacccagacc
caggcctagaccaagacctcgaccacggcctagaccccgaccaagacccaggcctagacctagaccaaggc
caagaccccgccctagacctagacctagacctaggcctcggcctagacctcggccagctagaatggtgagc
aagggcgaggagctgttcaccggggtggtgcccatcctggtcgagctggacggcgacgtaaacggccacaa
gttcagcgtgtccggcgagggcgagggcgatgccacctacggcaagctgaccctgaagttcatctgcacca
ccggcaagctgcccgtgccctggcccaccctcgtgaccaccctgacctacggcgtgcagtgcttcagccgc
taccccgaccacatgaagcagcacgacttcttcaagtccgccatgcccgaaggctacgtccaggagcgcac
catcttcttcaaggacgacggcaactacaagacccgcgccgaggtgaagttcgagggcgacaccctggtga
accgcatcgagctgaagggcatcgacttcaaggaggacggcaacatcctggggcacaagctggagtacaac
tacaacagccacaacgtctatatcatggccgacaagcagaagaacggcatcaaggtgaacttcaagatccg
ccacaacatcgaggacggcagcgtgcagctcgccgaccactaccagcagaacacccccatcggcgacggcc
ccgtgctgctgcccgacaaccactacctgagcacccagtccgccctgagcaaagaccccaacgagaagcgc
gatcacatggtcctgctggagttcgtgaccgccgccgggatcactctcggcatggacgagctgtacaagta
a
